# Targeting of tripartite neuron-cancer-immune cell crosstalk augments response to chemotherapy and immunotherapy

**DOI:** 10.1016/j.xcrm.2026.102994

**Published:** 2026-08-18

**Authors:** Dolly Jain, Nishant Pandey, Somesh K. Jha, Shreya Sinha, Ragini Singh, Junaid Alam, Bharti Aggarwal, Neelam Chauhan, Arunima Acharya, Anu P.V., Vandana Dhangar, Ritika Bhargava, Nikhil K. Chourasiya, Ali Khan, Arnab K. Sahoo, Debasish Nath, Lalita Mehra, Kajal Rana, Poonam Yadav, Jahanvi Ralhan, Saroj Rajput, Asish Pal, Arindam Maitra, Santiswarup Singha, Rajesh Panwar, Satish Khurana, Ujjaini Dasgupta, Sandeep K. Bhoriwal, Jyothi S. Prabhu, Prasenjit Das, Veena S. Patil, Avinash Bajaj

**Affiliations:** 1 Laboratory of Tumour Immune Microenvironment (TIME), Regional Centre for Biotechnology, 3^rd^ Milestone Faridabad Gurgaon Expressway, Faridabad 121001, India; 2 National Institute of Immunology, Aruna Asif Ali Marg, New Delhi 110067, India; 3 National Institute of Biomedical Genomics, Kalyani 741251, India; 4 School of Biology, Indian Institute of Science Education and Research Thiruvananthapuram, Thiruvananthapuram 695551, India; 5 Amity Institute of Integrative Sciences and Health, Amity University Haryana, Panchgaon, Manesar, Gurgaon 122413, India; 6 Institute of Nanoscience and Technology, Sector 81, Sahibzada Ajit Singh Nagar, Mohali 140306, India; 7 All India Institute of Medical Sciences, Ansari Nagar, New Delhi 110029, India; 8 ESIC Medical College and Hospital, New Industrial Town, Faridabad 121012, India; 9 Koita Centre for Digital Health, Trivedi School of Biosciences, Ashoka University, Sonipat 131029, India; 10 St. John’s Research Institute, 100 Feet Rd, John Nagar, Koramangala, Bengaluru 560034, India

**Keywords:** immunosuppression, colon cancer, cholinergic neurons, localized therapy, cancer therapy, hydrogel, acetylcholine receptors, tumor microenvironment

## Abstract

We report presence of cholinergic nerve fibers in the periphery and stroma of colon cancer tissues and their correlation with poor T cell and increased macrophage infiltration. We employed hydrogel-mediated localized delivery of an FDA-approved local anesthetic, bupivacaine (BUP), to target acetylcholine (ACh)-mediated crosstalk of cholinergic neurons with cancer and immune cells. Localized BUP-Gel therapy promotes T cell-mediated tumor inhibition and enhances the antitumor response of systemic chemotherapy and immunotherapy. Further, blockade of cancer- and immune cell-specific ACh receptors inhibits tumor growth, alters the TME, and augments the impact of chemotherapy and immunotherapy. Finally, we demonstrate that ACh receptor antagonists polarize macrophages toward an M1-like phenotype and activate T cell immunity in tumor explants of patients. Therefore, targeting cholinergic signals through localized delivery of anesthetics, as well as direct immune reprogramming via cholinergic receptor antagonists, may provide a means to modulate this tripartite crosstalk, with potential implications for therapeutic strategies.

## Highlights

• Cholinergic neurons are associated with immunosuppression in patients with colon cancer
• Acetylcholine suppresses cytotoxic T cell activity and M1 macrophage polarization
• Local anesthetics and receptor antagonists activate antitumor T cell immunity
• Targeting cholinergic signaling enhances chemotherapy and immunotherapy responses

## Graphical abstract

**Figure:**
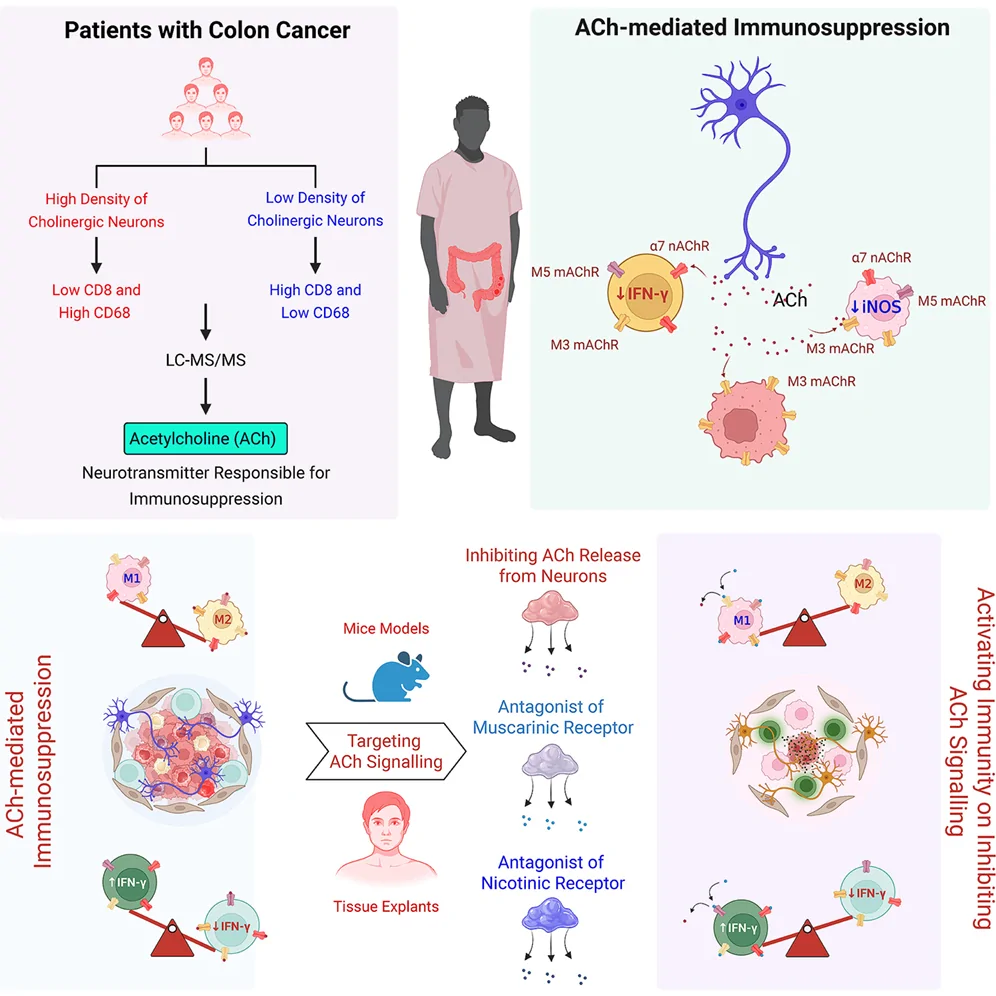

## Teaser

Jain et al. identify cholinergic nerve-derived acetylcholine as a mediator of immunosuppression in colon cancer and show that localized hydrogel-based delivery of local anesthetics and pharmacological blockade of acetylcholine receptors activate anti-tumor immune responses and enhance the efficacy of chemotherapy and immunotherapy.

## Introduction

Cancer cells sculpt their local microenvironment by utilizing growth signals and modulating host cells for their progression.^1^ This complex tumor microenvironment (TME) features immune cells, stromal cells, blood vessels, and extracellular matrix that coordinates with cancer cells, supporting their survival, growth, and spread.^2^ The interactions within TME, particularly between immune cells and cancer cells, create an immunosuppressive TME that diminishes the immune cell-mediated killing of cancer cells, thereby accelerating the tumor growth.^3^ Tumor innervation has emerged as a key hallmark of cancer progression, where innervating neurons facilitate immunosuppression along with cancer progression and metastasis.^4^^,^^5^ Tumor innervation is evident in solid tumors, including prostate, gastric, pancreatic, oral, breast, and skin,^6^^,^^7^^,^^8^^,^^9^^,^^10^ and the density of these nerve fibers has been associated with cancer-related pain and poor clinical outcomes.^11^^,^^12^ Neurons interact with cancer cells and other stromal cells, including immune cells in the TME primarily through neurotrophic factors, neuropeptides, and neurotransmitters that remodel the TME,^11^^,^^12^ thereby making it a promising therapeutic target for cancer treatment.^13^

Within the autonomic nervous system, sympathetic nerves contribute to tumor progression by releasing noradrenaline, which activates β-adrenergic receptors on cancer and stromal cells, thereby enhancing proliferation, angiogenesis, and immunosuppression.^9^^,^^14^^,^^15^ This adrenergic signaling also impairs CD8^+^ T cell function and promotes infiltration of tumor-associated macrophages (TAMs) and myeloid-derived suppressor cells (MDSCs).^16^^,^^17^ As a result, β-blockers are now being explored in clinical trials to inhibit adrenergic signaling in combination with conventional cancer therapies.^18^ Cholinergic signals from parasympathetic fibers promote tumor growth and metastasis via muscarinic receptor activation on cancer cells in prostate and gastric cancers, which upregulate nerve growth factor (NGF) and enhance parasympathetic nerve survival and function.^7^^,^^19^ In contrast, cholinergic signaling suppresses progression and stemness in pancreatic cancer.^20^ However, the role of cholinergic signaling in regulating immunosuppression in colon cancer is not known.

In addition to the sympathetic and parasympathetic nerves, highly complex and specialized enteric neurons present in the colon are organized as Meissner’s plexus and Auerbach’s plexus.^21^ Acetylcholine (ACh), one of the primary neurotransmitters released by these enteric neurons, maintains homeostasis in the colon.^22^ ACh maintains colonic homeostasis through interacting with specific muscarinic ACh and nicotinic ACh receptors (mAChR and nAChRs) that are known to be differentially expressed on different cell types.^23^^,^^24^ While cholinergic fibers releasing ACh are normally present in the muscularis propria as part of the enteric nervous system, their role in colon cancer progression and in the immunosuppressive TME is not known. Therefore, we hypothesize that ACh release from cholinergic neurons might play a critical role in tumor progression and its TME.

The effects of neurons can be targeted by either preventing neurotransmitter release or by blocking the signaling events initiated by the neurotransmitters.^13^ Local anesthetics, such as bupivacaine (BUP) and lidocaine, can impede the propagation of action potentials, thereby inhibiting the neurotransmitter release.^25^ Earlier studies showed that BUP inhibits the progression of breast and lung cancers,^26^^,^^27^ while pre-operative peritumoral infiltration of lidocaine enhances disease-free and overall survival in patients with breast cancer.^28^ Although earlier studies showed the impact of ACh receptor (mAChR and nAChR) antagonists as therapeutic interventions against many cancers,^29^^,^^30^ the consequences of targeting ACh-releasing nerve fibers or blocking ACh signaling in cancer and immune cells have not been studied. Therefore, we hypothesize that targeting ACh signaling or ACh release may suppress tumor growth by obstructing interactions between cholinergic neurons, cancer cells, and immune cells, ultimately modulating the TME. In this study, we showed the presence of cholinergic neurons in human colon cancer tissues and their tripartite cross-talk with TAMs and CD8^+^ T cells. We further demonstrated that targeting this ACh-mediated tripartite cross-talk of cholinergic neurons with cancer cells and immune cells inhibits tumor progression, mitigates immunosuppression, and activates T cell immunity.

## Results

### Cholinergic nerve fibers are associated with reduced CD8 T cells and increased macrophage infiltration in patients with colon cancer

**Figure 1:**
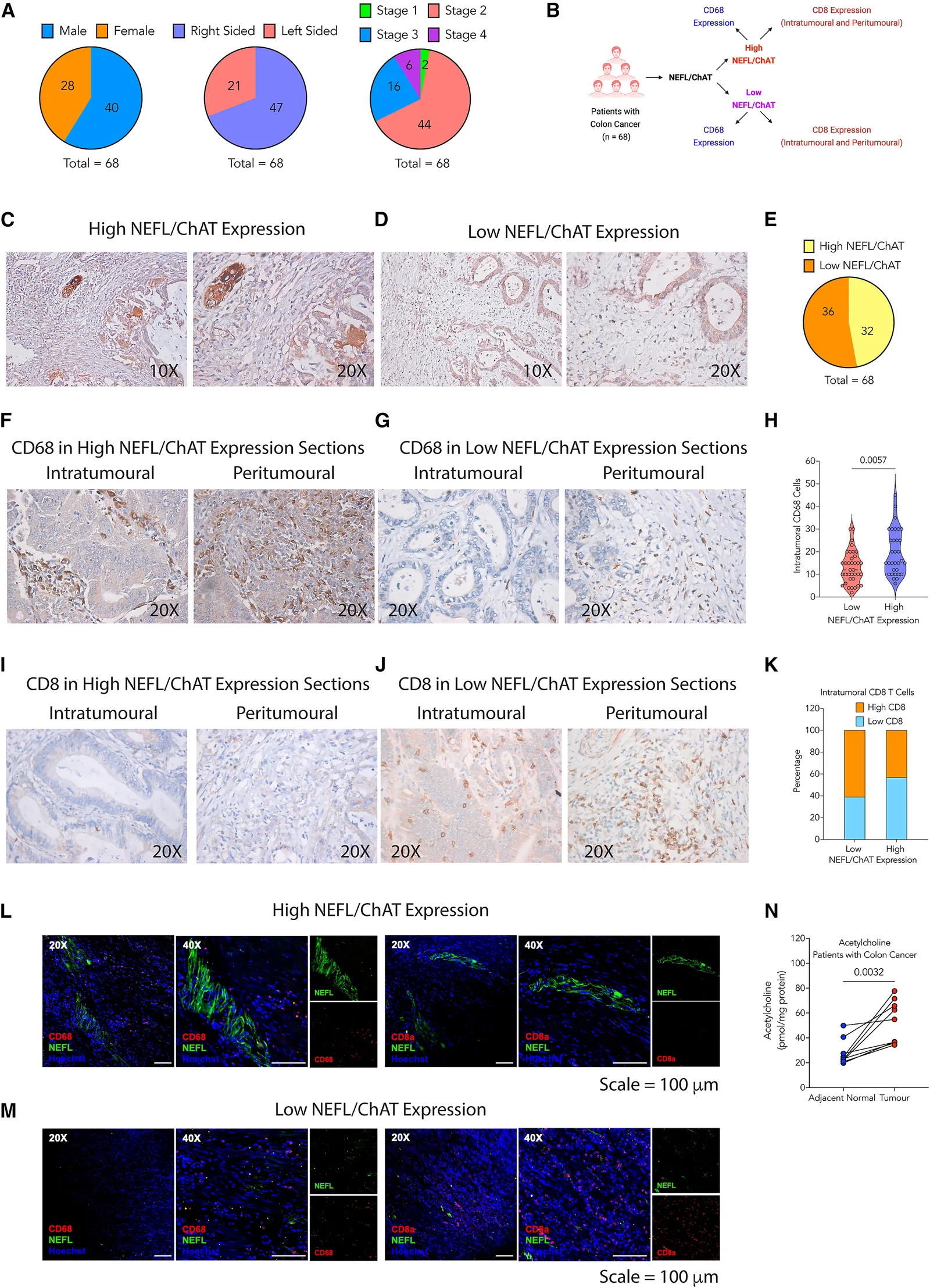
Presence of cholinergic nerve fibers is associated with reduced CD8 T cell and increased macrophage infiltration in patients with colon cancer (A) Pie charts showing the distribution of patients with colon cancer (*n* = 68) based on gender (male versus female), location of the tumor (right sided versus left sided) and stage which were studied for the correlation of the cholinergic neurons with macrophage and T cell infiltration. (B) Schematic showing the study design for immunohistochemistry (IHC) to investigate the correlation of the presence of cholinergic neurons with macrophage and T cell infiltration. (C and D) Representative IHC images of a tissue sections from patients with colon cancer with high (C) and low NEFL/ChAT expression (D) at different magnifications. (E) Pie chart showing the distribution of patients with high and low NEFL/ChAT expressing cholinergic neurons among patients with colon cancer. (F and G) Representative IHC images of CD68^+^ cells in tissue sections with high (F) and low (G) NEFL/ChAT expression in two patients with colon cancer. (H) Quantitation of CD68^+^ cells in the tissue sections indicates high CD68^+^ macrophages in sections with high NEFL/ChAT. (I and J) Representative IHC images of CD8^+^ cells in tissue sections with high (I) and low (J) NEFL/ChAT expression in two patients with colon cancer. (K) Percentage of CD8^+^ cells in the tissue sections for patients with stage II, indicating low CD8^+^ T cells in sections with high NEFL/ChAT. (L and M) Immunofluorescence images stained for NEFL, CD68, and CD8 in high (L) and low (M) NEFL/ChAT sections. (Scale bars, 100 μm). (N) Quantification of ACh in colon tumor tissues indicates the high levels of ACh compared to adjacent normal tissues (*n* = 10). See also Figure S1, Data S1, S2, and S3. Student’s *t* test for (H), Fisher’s exact test for (K) and paired Student’s *t* test for (N). ∗*p* < 0.05, ∗∗*p* < 0.001, and ∗∗∗∗*p* < 0.0001 for the indicated comparisons.

We used a cohort of 68 patients with colon cancer with distribution with respect to gender (male 40, female 28), location of tumor (47 right sided and 21 left sided) and stages (2 with stage I, 44 with stage II, 16 with stage III, and 6 with stage IV) (Figure 1A, Data S1). We stained the colon cancer tissue sections from these patients with neurofilament-L (NEFL), a general neuronal marker, and choline acetyltransferase (ChAT), a specific marker for cholinergic neurons (Figure 1B). The number of NEFL/ChAT-positive neurons was quantified in high-power fields (HPF) in tumor-dense and adjoining areas. We observed a high density of cholinergic nerve fibers, bundles, or ganglion cells within the tumor stroma and the tumor periphery in 32 of the 68 patients (∼47% of total patients) (Figures 1C–1E, Data S1). We further performed Kaplan-Meier survival analysis using the KM-Plotter database and found that concurrent high expression of both NEFL and ChAT is associated with worse overall survival (*p* = 0.0013) but not with recurrence-free survival (*p* = 0.062) (Figures S1A and S1B). We next analyzed a publicly available dataset (GSE14333) from patients with primary colorectal cancer and observed that high expression of NEFL and ChAT was associated with poor recurrence-free survival (*p* = 0.019) in early-stage colon cancer (Figure S1C and Data S2). Multivariate Cox regression analysis showed a hazard ratio (HR) of 1.64 (95% confidence interval, *p* = 0.02), suggesting that NEFL and ChAT expression may be independent prognostic factors in early stages (Figure S1D and Data S2). However, we did not observe a similar level of significance across all stages of colorectal cancer (HR = 1.37, *p* = 0.07), suggesting that NEFL and ChAT expression may be linked to disease progression and may have prognostic utility in early stages of colon cancer.

We next stratified the tumors into two groups based on median NEFL/ChAT expression: high (*n* = 32) and low (*n* = 36). We also quantified the average number of CD68^+^ macrophages across multiple HPFs (Figure 1B, Data S2). Tumors with high expression of NEFL/ChAT showed increased density of intratumoral CD68^+^ macrophages (Figures 1F–1H; Figures S1E and S1F). We then assessed infiltration of CD8^+^ T cells by counting the average number of CD8^+^ T cells across multiple HPFs and categorizing them as high or low based on a median cut-off in stage II samples (dataset 3). We observed low infiltration of CD8^+^ T cells in intratumoural regions in ∼57% of patients with high NEFL/ChAT expression, whereas there is an increased infiltration of CD8^+^ T cells in ∼61% of patients with low NEFL/ChAT expression (*p* = 0.0159) (Figures 1I–1K; Figures S1G and S1H). To validate these findings, we performed multiplex immunofluorescence analysis on patient samples stratified by high and low NEFL/ChAT expression. In samples with high NEFL/ChAT expression, we observed a higher frequency of CD68^+^ cells and a lower frequency of CD8^+^ T cells in regions surrounding nerve structures (Figure 1L). In contrast, samples with low NEFL/ChAT expression showed reduced abundance of CD68^+^ cells and a higher frequency of CD8^+^ T cells localized around nerve areas (Figure 1M). As ACh is the primary neurotransmitter released by cholinergic nerves, we quantified the ACh in colon cancer tissues using liquid chromatography-tandem mass spectrometry (LC-MS/MS). Notably, we observed a ∼2-fold higher level of ACh (*p* = 0.0032) in colon tumors compared with matched adjacent normal tissues (Figure 1N), suggesting a potential role for ACh in tumor progression. Therefore, the presence of cholinergic nerve fibers in colon cancer tissues is associated with increased macrophage recruitment and reduced infiltration of CD8^+^ cells, suggesting a potential immunosuppressive role for cholinergic signaling in the TME.

### ACh augments cancer cell proliferation

**Figure 2:**
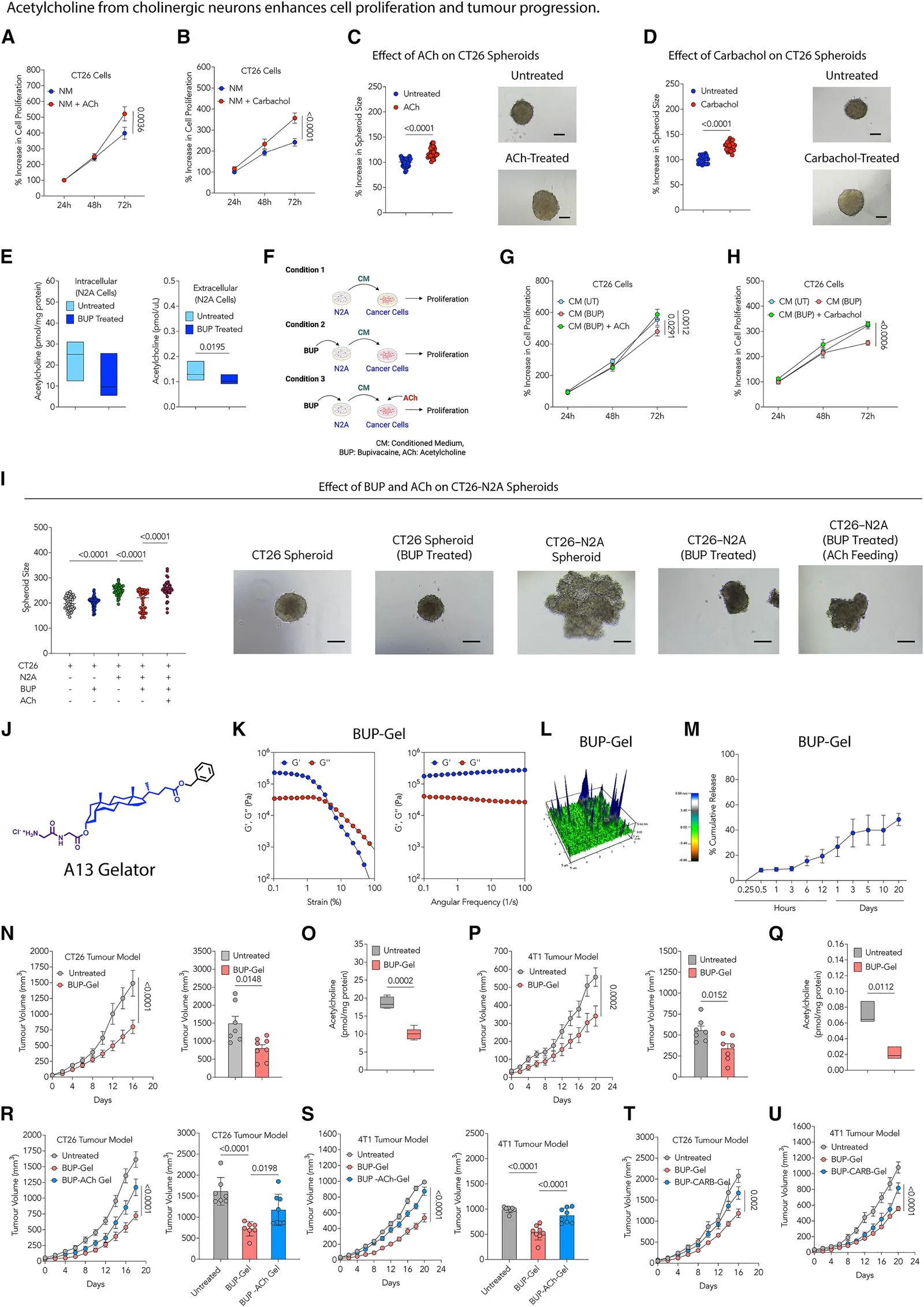
Localized delivery of bupivacaine, inhibiting ACh release, impedes tumor growth (A and B) Time-dependent change in the cell proliferation (mean ± SEM, *n* = 4) of CT26 cells on treatment with ACh (A) and carbachol (B). (C and D) Percentage increase in spheroid size (mean ± SEM, *n* = 3) and representative images of CT26 spheroids on treatment with ACh (C) and carbachol (D) (scale bars, 50 μm). (E) Quantification of intracellular and extracellular released ACh (mean ± SEM, *n* = 5–6) from untreated and BUP-treated N2A cells. (F) A schematic showing the study plan for deciphering the impact of conditioned media from untreated and BUP-treated N2A cells on cancer cell proliferation. (G and H) Change in cell proliferation (mean ± SEM, *n* = 3) of CT26 cells on treatment with conditioned media of untreated N2A cells (CM (UT)), conditioned media of BUP-treated N2A cells (CM (BUP)), ACh-fed conditioned media (G) and carbachol-fed (H) conditioned media of BUP-treated N2A cells. (I) Change in spheroid size (mean ± SEM, *n* = 3) and representative images of CT26 spheroids and N2A-CT26 co-spheroids on different treatments (scale bars, 50 μm). (J) Molecular structure of A13 gelator. (K) Amplitude and frequency sweep characterization of BUP-Gel. (L) AFM micrograph of BUP-Gel. (M) Release kinetics (mean ± SEM, *n* = 3) of BUP from BUP-Gel. (N and O) Tumor growth kinetics (mean ± SEM, *n* = 7–8) (N) and ACh quantification (O) (mean ± SEM, *n* = 4) in CT26 tumors on treatment with BUP-Gel. (P and Q) Tumor growth kinetics (mean ± SEM, *n* = 7–8) (P) and ACh quantification (Q) (mean ± SEM, *n* = 3) in 4T1 tumors on treatment with BUP-Gel. (R and S) Tumor growth kinetics (mean ± SEM, *n* = 7) and final day tumor volume of CT26 (R) and 4T1 (S) tumors on treatment with BUP-Gel and ACh-loaded BUP-ACh-Gel. (T and U) Tumor growth kinetics (mean ± SEM, *n* = 5–6) of CT26 (T) and 4T1 (U) tumors on treatment with BUP-Gel and carbachol-loaded BUP-CARB-Gel. See also Figure S2. Two-tailed Student’s *t* test for (A)–(E) and (N)–(Q). One-way ANOVA with Dunnett’s post-hoc test for (I) and (R)–(U) and two-way ANOVA with Tukey’s post-hoc test for (G), (H), (N), (P), and (R)–(U). ∗*p* < 0.05, ∗∗*p* < 0.001, and ∗∗∗∗*p* < 0.0001 for the indicated comparisons.

As our investigations on clinical samples revealed a higher incidence of cholinergic neurons and higher levels of ACh in colon tumor tissues compared to adjacent normal tissues, we hypothesized that ACh released by cholinergic nerve fibers may enhance cancer cell proliferation. Therefore, we determined the effect of ACh (50 μM) on cancer cell proliferation in a time-dependent manner. ACh supplementation led to a ∼1.3-fold increase in the proliferation of CT26 (murine colon cancer cell line) cells at 72 h in comparison to CT26 cells grown in a normal medium (NM) (Figure 2A). Similarly, there is a ∼1.1-fold increase in proliferation of 4T1 (murine breast cancer cell line) cells upon ACh supplementation at 72 h (Figure S2A). Similar to ACh, feeding of carbachol (an agonist of cholinergic receptors) (50 μM) also led to a ∼1.5-fold increase in the proliferation of CT26 cells and a ∼1.3-fold increase in the proliferation of 4T1 cells. (Figure 2B; Figure S2B). ACh feeding (50 μM) also led to a ∼1.2-fold increase in the size of CT26 spheroids and a ∼1.3-fold increase in the size of 4T1 spheroids compared to untreated spheroids (Figure 2C; Figure S2C). Similarly, feeding of carbachol (50 μM) showed a ∼1.2-fold increase in size of CT26 and 4T1 spheroids compared to untreated spheroids (Figure 2D; Figure S2D). Therefore, these results suggest that ACh and carbachol boost cancer cell proliferation, probably via activation of cholinergic receptors.

### BUP inhibits ACh-mediated cell proliferation

To validate the ACh-mediated crosstalk between cholinergic neurons and cancer cells, we employed BUP, an FDA-approved non-opioid, long-acting local anesthetic that blocks sodium and potassium channels on neurons and inhibits action potentials.^25^^,^^26^ We hypothesized that BUP-mediated inhibition of ACh release would disrupt ACh-driven interactions between cholinergic neurons and cancer cells, ultimately reducing cancer cell proliferation. To assess the effect of BUP, we first evaluated its cytotoxicity on Neuro2A (N2A) cells, a murine cholinergic neuroblast cell line. BUP treatment up to 100 μM did not affect the viability of N2A cells (Figure S2E). While BUP treatment did not change the intracellular ACh levels in N2A cells, there is a ∼1.3-fold decrease in extracellular ACh levels (Figure 2E). Culture of CT26 cells in the presence of BUP (100 μM)-treated N2A conditioned media (CM (BUP)) showed a ∼1.2-fold decrease in proliferation compared to CT26 cells treated with CM from untreated N2A cells (CM (UT)) (Figures 2F and 2G). Further, supplementation of ACh (50 μM) in CM (BUP) led to a ∼1.2-fold increase in cell proliferation (Figures 2F and 2G). Similarly, treatment of 4T1 cells with CM (BUP) resulted in a ∼1.3-fold decrease in proliferation, and ACh supplementation led to a ∼1.3-fold increase in proliferation (Figure S2F). Supplementation of carbachol (50 μM) also resulted in a ∼1.3- and a ∼1.4-fold increase in proliferation of CT26 and 4T1 cells in comparison to cancer cells that were treated with CM (BUP) (Figure 2H; Figure S2G). BUP treatment did not affect the cell viability of CT26 or 4T1 cells till 200 μM (Figures S2H and S2I). Supplementation of ACh did not alter the proliferation of BUP-treated N2A cells, suggesting that BUP downregulates ACh levels without affecting the viability or proliferation of N2A cells (Figure S2J). Moreover, BUP treatment did not affect the proliferation of N2A, CT26, and 4T1 cells (Figures S2J–S2L). In contrast, ACh supplementation increased proliferation by ∼1.2-fold in BUP-treated CT26 and 4T1 cells (Figures S2K and S2L), suggesting that BUP in the CM of N2A-treated cells is not responsible for inhibiting cancer cell proliferation. We also quantified ACh levels in CT26 and 4T1 cells treated with BUP and observed ∼4-fold and ∼2-fold decreases in intracellular ACh levels, which may be due to BUP’s non-specific actions (Figures S2M and S2N). However, there was no significant change in the released ACh from BUP-treated CT26 and 4T1 cells (Figures S2M and S2N), suggesting that BUP treatment selectively attenuates ACh release in neuronal cells without affecting the extracellular ACh levels in cancer cells.

We next created co-spheroids of CT26 and 4T1 cells with N2A cells at a 9:1 ratio and observed an increase in spheroid size compared to CT26 and 4T1 spheroids alone (Figure 2I; Figure S2O). BUP treatment did not affect the spheroid size of CT26 spheroids, whereas BUP-treated co-spheroids (CT26-N2A) showed a ∼1.2-fold decrease compared to untreated co-spheroids (Figure 2I). There was a ∼1.2-fold increase in spheroid size upon ACh feeding to BUP-treated co-spheroids compared to only BUP-treated co-spheroids (Figure 2I). Similarly, BUP-treated 4T1/N2A co-spheroids showed a ∼1.3-fold decrease in spheroid size compared to untreated 4T1 co-spheroids, and ACh feeding mitigated this effect (Figure S2O). Therefore, these results demonstrate that BUP inhibits ACh-mediated crosstalk between cholinergic neurons and cancer cells, thereby inhibiting cancer cell proliferation.

### Localized delivery of BUP inhibits tumor progression

As local anesthetics need to be delivered at their action site, and any systemic exposure can cause toxicity,^31^ we hypothesize that hydrogel-mediated localized delivery of BUP would be an effective strategy to inhibit the release of ACh from peripheral cholinergic neurons. In addition, the hydrogel-mediated sustained release of BUP will provide reduced toxicity compared to carrier-free delivery. In our earlier studies, we reported the engineering of a non-immunogenic, lithocholic acid-derived, low-molecular-weight hydrogelator (A13) (Figure 2J).^32^ We demonstrated that A13 hydrogel can entrap a range of chemotherapeutic drugs without altering the physical properties of the gel and allow the sustained and sequential release of a combination of chemotherapeutic drugs.^32^^,^^33^ A13 hydrogel scaffold exhibited good biocompatibility, *in vivo* persistence with minimal immune infiltration, and gradual clearance over 21–50 days in rodents.^32^^,^^33^ We hypothesized that entrapment of BUP in the A13 hydrogel would allow us to implant the BUP-loaded gel (BUP-Gel) near the tumor site and inhibit tumor progression by mitigating ACh release. Therefore, we loaded BUP into an A13 gel by mixing ∼4 mg of BUP with ∼70 mg of the A13 gelator in 1 mL of water. Heating the mixture yielded a clear solution, and subsequent cooling led to the formation of BUP-Gel. Comparative rheological investigation showed a slight increase in the mechanical rigidity of the BUP-Gel relative to the native A13 gel, as evident by the elastic moduli and yield stresses, presumably due to drug-gelator interactions. (Figure 2K; Figure S2P). Atomic force micrographs indicated that surface roughness decreased upon the incorporation of BUP compared to only A13 neat gel (Figure 2L; Figure S2Q). Drug release kinetics from BUP-Gel suggested a sustained release of BUP from A13 gel over time (Figure 2M). We next evaluated self-healing behavior and structural stability upon exposure to deformation and observed that both A13-Gel and BUP-Gel exhibited good recovery (Figure S2R). Thermal stability analysis revealed a slight reduction in the storage modulus without any change in the loss modulus in both A13-Gel and BUP-Gel (Figure S2S), indicating maintenance of gel integrity and suggesting the good thermal stability of the hydrogels.

As the neuro-immuno-cutaneous system releasing ACh is the key component of skin,^34^ we tested the impact of BUP-Gel on tumor growth kinetics against subcutaneous syngeneic murine colon (CT26), breast (4T1), and melanoma (B16) cancer models, where tumor-bearing mice were either left untreated or treated with a single dose of BUP-Gel (40 mg/kg of BUP). Tumor growth kinetics revealed a significantly slower rate of progression on BUP-Gel treatment in CT26 tumors compared to untreated tumors with a ∼2-fold decrease in ACh levels (Figures 2N and 2O). Similarly, BUP-Gel-treated 4T1 tumors showed reduced tumor growth kinetics, with a 3.5-fold decrease in ACh levels (Figures 2P and 2Q), suggesting that BUP-Gel inhibits tumor growth, likely by reducing ACh release. We validated a similar finding in the B16 tumor model (Figures S2T and S2U). To validate that inhibition of ACh is responsible for slow tumor growth kinetics, we prepared an ACh-loaded BUP-Gel (BUP-ACh-Gel) that can release the ACh along with BUP and compared the tumor growth kinetics of BUP-Gel with BUP-ACh-Gel against CT26, 4T1, and B16 tumor models. Although BUP-Gel showed a ∼2-fold reduction in tumor growth at day 16, there was a ∼1.7-fold increase in tumor volume upon BUP-ACh-Gel treatment compared to BUP-Gel-treated CT26 tumors (Figure 2R). BUP-ACh-Gel also showed a higher tumor growth rate than BUP-Gel in the 4T1 (Figure 2S) and B16 models (Figure S2V). Similarly, BUP-Gel loaded with carbachol (BUP-CARB-Gel) enhanced the tumor growth kinetics in CT26 and 4T1 models compared with BUP-Gel (Figures 2T and 2U). Therefore, these results suggest that localized delivery of BUP inhibits ACh, thereby mediating tumor growth inhibition.

### BUP-Gel therapy alters the immune landscape in tumor tissues

**Figure 3:**
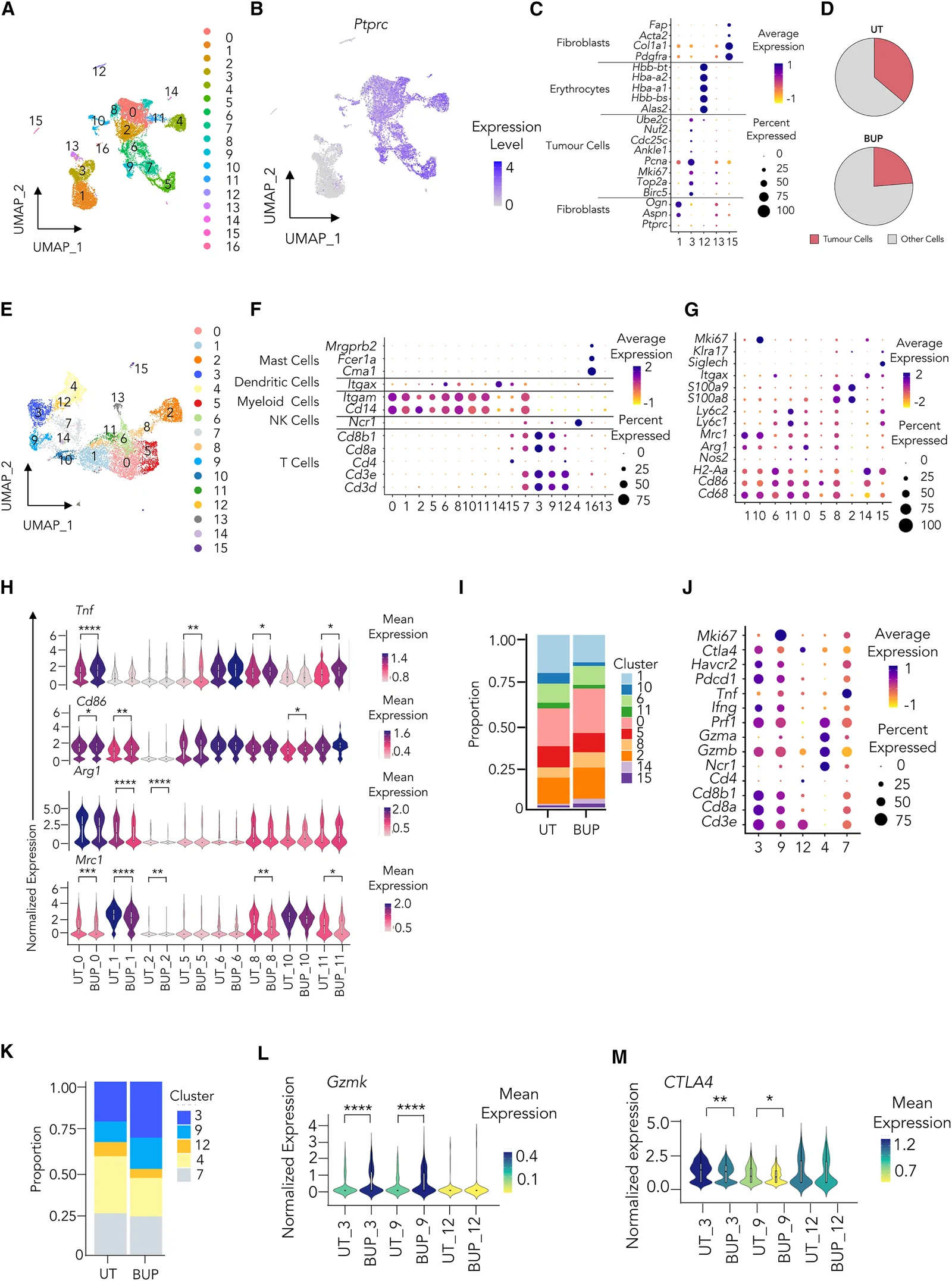
BUP-Gel therapy alters the immune landscape in tumor tissues (A) UMAP from 13,939 cells shows sixteen distinct clusters of cells. (B) UMAP plot showing expression of *Ptprc*. The scale indicates the expression of the specified transcript. Cells with zero counts are displayed in gray. (C) Dot plot showing the average expression (color) and the proportion of cells expressing each selected gene transcript (dot size) across non-immune cell clusters. (D) Proportion of tumor cells within CD45^−^ cells in UT and BUP-treated groups. (E) The UMAP plot displays sixteen distinct clusters consisting of all immune cells after re-clustering. (F) Dot plot representing the average expression (color) and the proportion of cells expressing each selected gene transcript (dot size) across immune cell clusters. (G) Dot plot representing the average expression (color) and the proportion of cells expressing each selected gene transcript (dot size) across myeloid and dendritic cell clusters. (H) Violin plots showing the expression of transcripts *Tnf* (H), *Cd86* (I), *Arg1* (J), and *Mrc1* (K) within different myeloid cell clusters from UT and BUP-treated groups. The color scale shows the mean expression of the indicated transcript. (I) Proportion of different myeloid cell clusters in UT and BUP-treated groups. (J) Dot plot representing the average expression (color) and the proportion of cells expressing each selected gene transcript (dot size) across T and NK cell clusters. (K) Proportion of cells in different T and NK cell clusters in UT and BUP-treated groups. (L and M) Violin plots showing the expression of *Gzmk* (L) and *Ctla4* (M) transcripts within different T cell clusters from UT and BUP-treated groups. The color scale shows the mean expression of the indicated transcript. See also Figure S3. Two-tailed Student’s *t* test for (H), (L), and (M). ∗*p* < 0.05, ∗∗*p* < 0.001, and ∗∗∗∗*p* < 0.0001 for the indicated comparisons.

As we observed a correlation of cholinergic neurons with macrophage and T cell infiltration in patients with colon cancer, we investigated the impact of BUP-Gel therapy, which inhibits ACh release, on the tumor immune microenvironment. We performed single-cell RNA sequencing (scRNA-seq) on untreated (UT) and BUP-Gel-treated CT26 tumors using the 10X genomics platform (Figure S3A).^35^ Unsupervised clustering of 13,939 QCed cells, obtained from UT and BUP-Gel-treated tumor samples, identified 16 distinct clusters (Figure 3A; Figure S3B). We classified the clusters as immune cell clusters (0, 2, 4, 5, 6, 7, 8, 9, 10, 11, 14, and 16) and non-immune cell clusters (1, 3, 12, 13, and 15) based on the *Ptprc* expression (Figure 3B). The non-immune cells mainly consisted of fibroblasts, cancer cells, and erythrocytes, with clusters 1 and 15 showing higher expression of fibroblast-associated transcripts (Figure 3C) and cluster 12 showing erythrocyte-specific transcripts (Figure 3C). Cluster 3 showed high levels of transcripts associated with the cell cycle, such as *Mki67*, *Top2a*, *Pcna*, and *Birc5*, which were classified as proliferating tumor cells (Figure 3C). Notably, we observed a reduced proportion of proliferating tumor cells (cluster 3) in BUP-treated tumors compared to UT tumors, supporting that BUP inhibits tumor growth (Figure 3D).

We re-clustered the *Ptprc*^*+*^ immune cell clusters to identify the finer details of various sub-populations of immune cells (Figure 3E; Figure S3C). These sixteen clusters could be grouped into major immune populations, such as myeloid cells expressing *Cd14 and Itgam* (clusters 0, 1, 2, 5, 6, 7, 8, 10, and 11), dendritic cells expressing *Itgax* (clusters 6, 14, 15), T cells expressing *Cd3* (clusters 3, 7, 9, and 12), NK cells expressing *Ncr1* (cluster 4), and mast cells expressing *Mrgprb2*, *Fcer1a*, and *Cma1* (cluster 16) (Figure 3F). All myeloid cell clusters were segregated into specific cell types, including monocytes characterized by *Ly6c1* and *Ly6c2* expression (cluster 5), and intermediate macrophages that express *Cd68* and *Ly6c1/Ly6c2* (clusters 0 and 11). MDSCs were defined by high expression of *S100a8* and *S100a9* (clusters 2 and 8) (Figure 3G). In addition, dendritic cell subsets were classified into classical dendritic cells (cDCs) expressing *Itgax* (cluster 14), monocyte-derived dendritic cells (moDCs) expressing *Cd14* and *Itgax* (cluster 6), and plasmacytoid dendritic cells (pDCs) expressing *Siglech* and *Klra17* expression (cluster 15) (Figure 3G).

To delineate the macrophage heterogeneity, we analyzed the expression of genes associated with M1-like macrophages (*Cd86* and *Tnf)* and M2-like macrophages (*Mrc1* and *Arg1*). Cluster 1 showed a distinct M2-like profile with high *Mrc1* and *Arg1*, and cluster 10 displayed high proliferative capacity (*Mki67*) in addition to M2-like features (Figure 3G). In contrast, cluster 11 expressed M1-like markers (*Cd86* and *Nos2*); cluster 0 showed a mixed M1/M2 profile (*Nos2*, *Arg1*, and *Mrc1*); and cluster 8 (monocytic MDSCs) expressed *Mrc1*, *Arg1*, and *Cd86*, indicating a hybrid state (Figure 3G). Comparison of the expression of M1-like (*Tnf* and *Cd86*) and M2-like (*Arg1* and *Mrc1*) markers across macrophages, MDSCs and monocytic clusters in UT and BUP-treated tumors revealed elevated *Tnf* expression in cluster 11 in BUP-treated tumors, consistent with their M1-like phenotype (Figure 3H). Elevated *Tnf* was also observed in clusters 0, 5, and 8 upon BUP treatment (Figure 3H). *Cd86* expression was found to be increased in M1/M2 hybrid macrophages (cluster 0) and was also high in other populations, including M2-like macrophages (clusters 1 and 10) (Figure 3H). In contrast, the expression of M2 marker, *Arg1*, decreased in M2-like macrophages (cluster 1) as well as in MDSCs (cluster 2) upon BUP treatment (Figure 3H). Similarly, *Mrc1* expression was reduced in M2-like macrophages (clusters 1), M1-like macrophages (cluster 11), M1/M2 hybrids (cluster 0), and monocytic MDSCs (cluster 8) (Figure 3H). We further compared the proportion of cells within myeloid cell clusters in UT and BUP-Gel-treated tumors and observed a reduced proportion of M2-like macrophages (clusters 1 and 10) in BUP-Gel-treated tumors (Figure 3I).

Among T cell populations, *Cd8*-expressing cells (clusters 3, 7, and 9) are identified as cytotoxic T cells, while cluster 12 expressing *Cd4* is classified as a helper T cell cluster (Figure 3J). Among CD8^+^ subsets, cluster 3 showed elevated expression of cytotoxic markers (*Gzmb*, *Gzmk*, *Prf1*, and *Ifng*) and immune checkpoints (*Pdcd1*, *Havcr2*, and *Ctla4*), indicating an activated phenotype (Figure 3J). Cluster 9 also expressed cytotoxic markers and immune checkpoints with additional expression of *Mki67*, suggesting proliferative capacity (Figure 3J). CD4^+^ T cells in cluster 12 expressed high *Ctla4*, and NK cells (cluster 4) exhibited expression of *Gzmb* and *Prf1* (Figure 3J). BUP treatment led to an increase in CD8^+^ T cells (clusters 3 and 9) and a reduction in *Ctla4*-expressing CD4^+^ T cells (cluster 12) (Figure 3K). Comparison of cytotoxic marker and immune checkpoint expression across T cell clusters revealed that *Gzmk* was upregulated in CD8^+^ T cells (clusters 3 and 9) following BUP treatment (Figure 3L), while *Ctla4* expression was decreased across all T cell clusters in BUP-treated tumors (Figure 3M). Collectively, these findings suggest that BUP modulates the TME by shifting the tumor-associated myeloid compartment toward an M1-like phenotype and enhancing cytotoxic T cell activity, thereby contributing to immune activation and anti-tumor immune responses.

### Localized BUP-Gel therapy polarizes macrophages toward the M1 type

**Figure 4:**
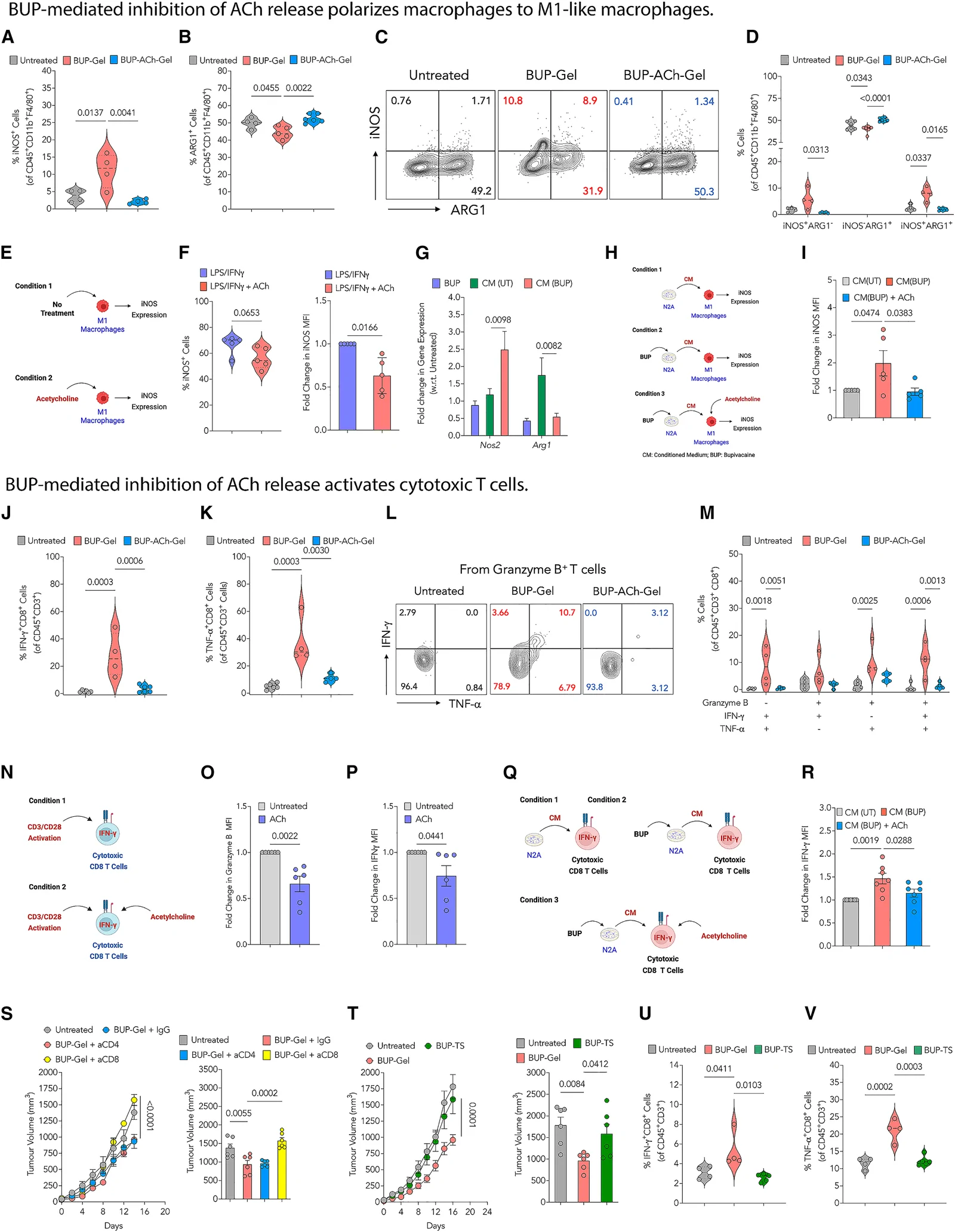
Localized BUP-Gel therapy polarizes macrophages toward the M1 type and activates T cells (A and B) Change in the population (mean ± SEM, *n* = 4–5) of iNOS^+^F4/80^+^ M1-like macrophages (A) and ARG1^+^F4/80^+^ M2-like macrophages (B) in untreated, BUP-Gel-treated and BUP-ACh-Gel-treated CT26 tumors. (C and D) Flow cytometry plots (C) and the percentage change in F4/80^+^ macrophages (D) (mean ± SEM, *n* = 4) expressing only iNOS (iNOS^+^ARG1^-^), only ARG1 (iNOS^−^ARG1^+^), and both iNOS and ARG1 (iNOS^+^ARG1^+^) F4/80^+^ macrophages in untreated, BUP-Gel-treated and BUP-ACh-Gel-treated CT26 tumors. (E) A schematic showing the study plan to determine the effect of ACh on LPS/IFN-γ-induced M1-like BMDMs. (F) Percentage change (mean ± SEM, *n* = 5) in iNOS^+^ macrophages and iNOS MFI on treatment with ACh. (G) Fold-change in the gene expression (mean ± SEM, *n* = 3) of *Nos2* and *Arg1* in M0 BMDMs on treatment with conditioned media of untreated N2A cells (CM (UT)) and BUP-treated N2A cells (CM (BUP)). (H) A schematic showing the study plan to decipher the effect of CM (UT), CM (BUP), CM(BUP)+ACh on LPS, and IFN-γ-induced M1-like BMDMs. (I) Percentage change (mean ± SEM, *n* = 5) in iNOS^+^ macrophages and iNOS MFI on treatment with CM (UT), CM (BUP), and CM(BUP)+ACh. (J and K) Change in the population (mean ± SEM, *n* = 4–6) of IFN-γ^+^CD8^+^ (J) and TNF-α^+^CD8^+^ (K) cells in untreated, BUP-Gel-treated, and BUP-ACh-Gel-treated CT26 tumors. (L and M) Flow cytometry plots (L) and percentage change in the CD8^+^ populations (M) (mean ± SEM, *n* = 4) co-expressing granzyme B, IFN-γ, or TNF-α in untreated, BUP-Gel-treated and BUP-ACh-Gel-treated CT26 tumors. (N) A schematic showing the study plan to determine the effect of ACh on stimulated mouse splenic CD8^+^ T cells. (O and P) Change (mean ± SEM, *n* = 6) in granzyme B MFI (O) and IFN-γ MFI (P) in CD8 T cells on treatment with ACh. (Q) A schematic showing the study plan to decipher the effect of CM (UT), CM (BUP), and CM (BUP) + ACh on stimulated murine CD8 T cells. (R) Change in the IFN-γ MFI (mean ± SEM, *n* = 7) of CD8 T cells on treatment with CM (UT), CM (BUP), and CM(BUP)+ACh. (S) Tumor growth kinetics and final day tumor volume (mean ± SEM, *n* = 6) of CT26 tumors on treatment with BUP-Gel with isotype IgG control, BUP-Gel with anti-CD4 antibody, and BUP-Gel with anti-CD8 antibody. (T) Tumor growth kinetics (mean ± SEM, *n* = 6) in untreated, BUP-Gel-treated, and BUP-TS-treated CT26 tumors. (U and V) Percentage change in the population (mean ± SEM, *n* = 4) of IFN-γ^+^CD8^+^ (U) and TNF-α^+^CD8^+^ (V) cells in untreated, BUP-Gel-treated, and BUP-TS-treated CT26 tumors. See also Figure S4 and Table S1. One-way ANOVA with Dunnett’s post-hoc test for (A), (B), (D), (G), (I), (J), (K), (M), (R), (U), and (V), Two-tailed Student’s *t* test for (F), (O), and (P) and two-way ANOVA with Tukey’s post-hoc test for (S) and (T). ∗*p* < 0.05, ∗∗*p* < 0.001, and ∗∗∗∗*p* < 0.0001 for the indicated comparisons.

Our scRNA-seq analysis (untreated versus BUP-Gel-treated tumors) revealed pronounced transcriptional alterations in macrophages and cytotoxic T cells upon BUP-Gel treatment. As we did not observe marked changes in other immune and stromal cell populations, we focused our further studies on macrophages and cytotoxic T cells, as these subsets exhibited the most substantial BUP-induced remodeling and are known to be crucial players of tumor immunity. As scRNA-seq results revealed that BUP-Gel therapy induces reprogramming of macrophages within the TME,^36^ we performed flow cytometry analysis for M1-like (iNOS^+^) and M2-like (ARG1^+^) macrophages in CT26 tumors following BUP-Gel treatment (Figure S4A). To validate whether these effects were mediated through ACh, we also included the tumors treated with BUP-ACh-Gel therapy. We observed a ∼3-fold increase in iNOS^+^ macrophages in BUP-Gel-treated tumors compared to untreated tumors (Figure 4A). Interestingly, ACh abrogated this effect by ∼5-fold reduction in iNOS^+^ macrophages when compared with BUP-Gel-treated tumors, suggesting that inhibition of ACh release on BUP-Gel therapy is responsible for the polarization toward M1-like macrophages (Figure 4A). BUP-Gel treatment also caused a ∼1.1-fold decrease in ARG1^+^ macrophages compared with untreated tumors, and ACh mitigated this effect (Figure 4B). Macrophage polarization in the TME cannot be fully captured by a simple M1 versus M2 classification but rather reflects a mixed or hybrid M1/M2-like profile. Therefore, we further analyzed macrophage populations expressing either iNOS alone, ARG1 alone, or both markers. BUP-Gel therapy led to a >3-fold increase in iNOS^+^ARG1^-^ macrophages, a ∼4-fold increase in iNOS^+^ARG1^+^ macrophages and a ∼1.1-fold reduction in the frequency of iNOS^−^ARG1^+^ macrophages compared to untreated tumors and these changes were abolished in BUP-ACh-Gel-treated tumors (Figures 4C and 4D). Our scRNA-seq results revealed similar heterogeneous macrophage populations with overlapping expression of canonical M1 and M2 markers, consistent with a spectrum of polarization states.

To validate the effect of ACh on macrophage polarization, we converted murine bone marrow-derived macrophages (BMDMs) into M1-like phenotype using LPS and IFN-γ followed by ACh (50 μM) treatment (Figure 4E; Figure S4B). ACh treatment led to a ∼1.1-fold decrease in iNOS^+^ cells with a ∼1.6-fold decrease in iNOS MFI (mean fluorescence intensity) in comparison to untreated M1-like BMDMs, suggesting the suppressive role of ACh on M1 polarization (Figure 4F). To further validate the ACh-mediated neuron-macrophage interactions, we treated BMDMs with conditioned media of untreated (CM-UT) and BUP-treated N2A cells (CM-BUP) and examined the *Nos2* and *Arg1* expression by quantitative reverse-transcription PCR (RT-qPCR) (Table S1). BMDMs treated with CM (UT) exhibited a ∼1.7-fold increase in *Arg1* expression compared to untreated BMDMs, while CM (BUP) treatment displayed a >3-fold decrease in *Arg1* expression compared with CM (UT)-treated BMDMs (Figure 4G). Interestingly, BMDMs treated with CM (BUP) showed a ∼2-fold increase in *Nos2* expression in comparison to BMDMs treated with CM (UT) (Figure 4G). Further, we treated M1-like BMDMs with CM (UT) and CM (BUP) and evaluated iNOS expression by flow cytometry (Figure 4H). CM (BUP) treatment led to a ∼2-fold increase in iNOS MFI in comparison to M1-like BMDMs treated with CM(UT), whereas iNOS expression was reversed upon treatment of M1-like BMDMs with ACh-supplemented CM(BUP) (Figure 4I). Therefore, consistent with our scRNA-seq results, these findings suggest that inhibiting ACh-mediated signaling promotes the polarization toward an M1-like macrophage phenotype.

### Localized BUP-Gel therapy activates the cytotoxic T cell response

As scRNA-seq results showed an increase in the proportion of CD8^+^ T Cells in BUP-Gel-treated tumors,^37^ we profiled the T cells for cytotoxic markers using flow cytometry in CT26 tumors treated with BUP-Gel and BUP-ACh-Gel (Figure S4C). BUP-Gel treatment led to a >25-fold increase in IFN-γ^+^CD8^+^ T cells compared to untreated CT26 tumors (Figure 4J). However, ACh abrogated this effect and caused a >8-fold decrease in IFN-γ^+^CD8^+^ T cells (Figure 4J). We observed a ∼7.5-fold increase in TNF-α^+^CD8^+^ T cells in BUP-Gel-treated tumors compared to untreated tumors, and ACh mitigated this effect (Figure 4K). We next assessed the co-expression of granzyme B with cytokines IFN-γ and TNF-α in CD8^+^ T cells and observed a marked increase in CD8^+^ T cells co-expressing IFN-γ and TNF-α, granzyme B and TNF-α, as well as granzyme B, IFN-γ, and TNF-α upon BUP-Gel treatment, and ACh abrogated this effect (Figures 4L and 4M). Recent studies have shown that CD4^+^ T cells, like CD8^+^ T cells, exert cytotoxic functions by releasing granzymes and effector cytokines, often in response to viral infections and cancer.^38^ Our results showed a ∼7-fold increase in IFN-γ^+^CD4^+^ T cells compared to untreated CT26 tumors, and ACh abrogated this effect (Figure S4D). We also observed a ∼13-fold increase in TNF-α^+^CD4^+^ T cells in BUP-Gel-treated tumors, and ACh feeding decreased it by ∼3.5-fold (Figure S4E). Analysis of exhaustion markers (PD1, TIM3, and CTLA4) on T cells revealed that BUP-Gel treatment resulted in a ∼2-fold decrease in PD1^+^TIM3^-^ CD8^+^ T cells, with no significant change in PD1^+^TIM3^+^ CD8^+^ T cells, and a ∼5-fold increase in CTLA4^+^ CD4^+^ T cells compared to untreated cells (Figures S4F–S4H). Interestingly, ACh further enhanced the expression of exhaustion markers, increasing PD1^+^TIM3^-^ and PD1^+^TIM3^+^CD8^+^ T cells, as well as CTLA4^+^ CD4^+^ T cells, suggesting that ACh not only suppresses cytotoxic markers but also promotes T cell exhaustion, thereby keeping a check on the T cell activation (Figures S4F–S4H).

To validate the ACh-mediated effect on T cell cytotoxicity, we stimulated mouse splenic CD8^+^ T cells and treated them with ACh (150 μM) (Figure 4N; Figure S4I). ACh treatment led to a ∼1.6-fold decrease in MFI of granzyme B and a ∼1.4-fold decrease in the MFI of IFN-γ compared to untreated CD8^+^ T cells (Figures 4O and 4P). We further investigated ACh-mediated neuron-T cell interactions, where stimulated T cells were treated with conditioned media from untreated (CM-UT) or BUP-treated (CM-BUP) N2A cells (Figure 4Q). We observed a ∼1.5-fold increase in IFN-γ MFI in CM(BUP) treated CD8^+^ T cells compared to CM (UT) treated T cells, which decreased in ACh-supplemented CM (BUP) treated CD8^+^ T cells (Figure 4R). These results indicate that ACh-mediated signaling suppresses cytotoxic T cell responses, and its inhibition enhances T cell activation.

Next, to delineate the impact of CD4 and CD8 T cells, we depleted CD4^+^ and CD8^+^ T cells in BUP-Gel-treated CT26 tumor-bearing mice to assess tumor inhibition. Interestingly, depletion of CD8^+^ T cells abrogated the effect of BUP-Gel therapy on tumor volume (Figure 4S; Figure S4J). However, we did not observe any change in tumor volume upon CD4^+^ T cell depletion. Therefore, although BUP-Gel therapy induces both CD8^+^ and CD4^+^ cytotoxic T lymphocytes (CTLs), the magnitude of cytolytic activity of CD8^+^ CTLs is markedly superior to that of the CD4^+^ CTLs. This does not exclude the meaningful contributions of CD4^+^ CTLs and other immune effector cells in building an effective anti-tumor immune network when treated with BUP-Gel. We also investigated the effect of BUP-Gel therapy in immunocompromised mice (NSG) using the CT26 tumor model and did not observe any change in tumor growth compared to BALB/c mice (Figure S4K).

We next assessed central memory and effector memory T cells in the peripheral blood of mice bearing CT26 tumors to determine the impact of BUP-Gel on these memory T cell subsets (Figure S4L). BUP-Gel treatment led to ∼2.5-fold increase in central memory T cells and ∼1.5-fold increase in effector memory T cells (Figures S4M and S4N). To investigate the effect of BUP-Gel on long-term memory T cells, we resected CT26 tumors at 50 mm^3^ and treated the resection site with BUP-Gel. We observed that 3 out of 8 mice did not show tumor recurrence. After 20 days of BUP-Gel treatment, we rechallenged these three mice with CT26 cells and observed a ∼4-fold slower rate of tumor growth compared to naive untreated mice at day 36 (Figure S4O). Upon assessing long-term memory cells in peripheral blood at day 36, we observed a >3.5-fold increase in central memory CD8 T cells in BUP-Gel-treated tumors, without any change in effector memory T cells, compared to untreated tumors (Figures S4P and S4Q).

Since we employed hydrogel-mediated delivery of BUP to delineate changes in the immune microenvironment, we investigated whether BUP-Gel offers advantages over the direct tumor-site (BUP-TS) injection of BUP without hydrogel. We observed that BUP-TS did not significantly affect tumor progression (Figure 4T). Although BUP-TS did not induce any change in IFN-γ^+^CD8^+^ T cells and TNF-α^+^CD8^+^ T cells, there was a similar decrease in MFI of ARG1 within macrophages (Figures 4U and 4V; Figure S4R). Therefore, these results suggest that BUP-Gel outperforms BUP-TS in inhibiting tumor growth and modulating the TME, primarily due to the sustained release of BUP from A13 gel.

### Localized BUP-Gel therapy augments the efficacy of systemic chemotherapy and immunotherapy

**Figure 5:**
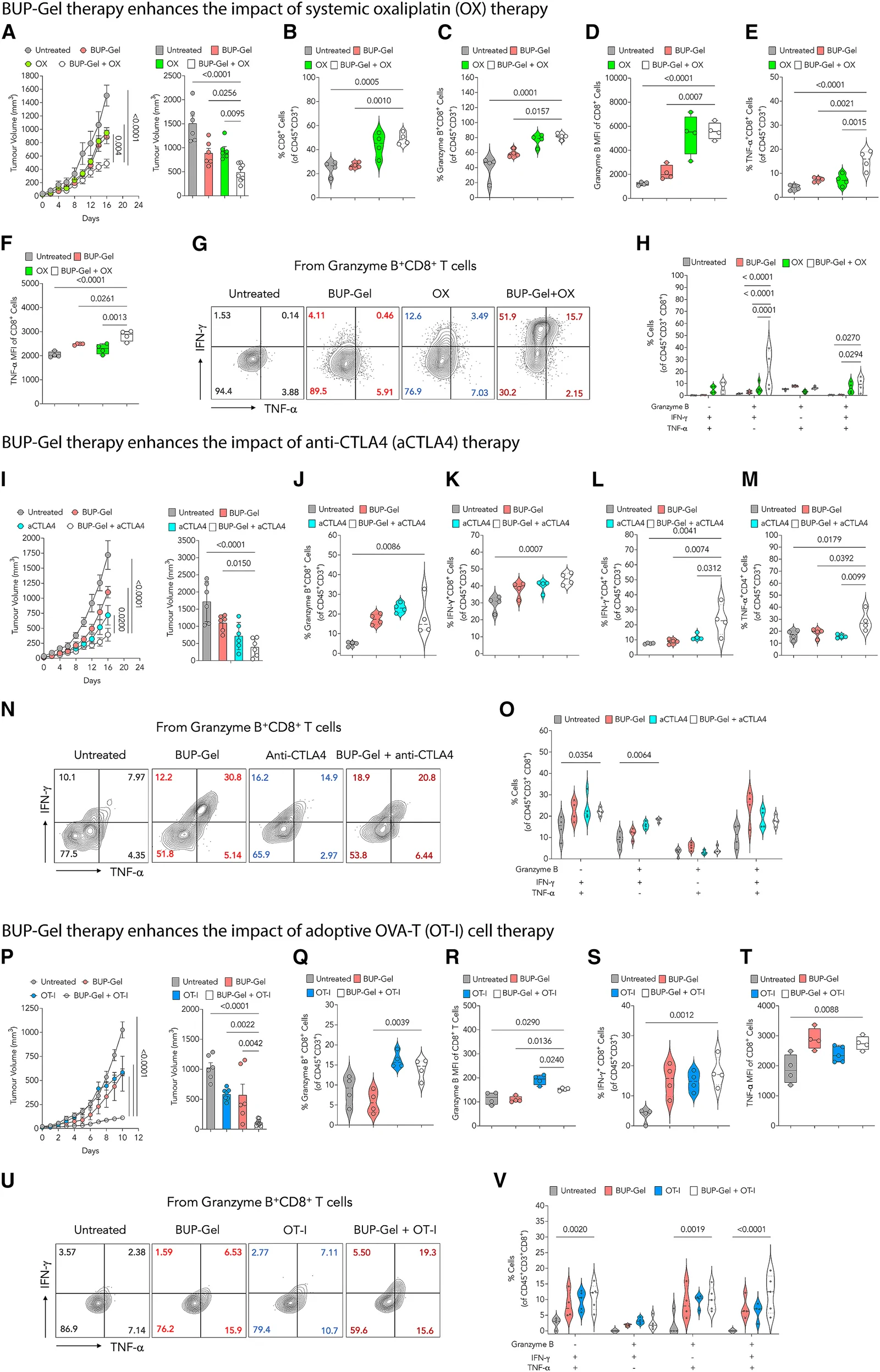
BUP-Gel therapy augments the effect of systemic chemotherapy and immunotherapy (A) Tumor growth kinetics and final day tumor volume (mean ± SEM, *n* = 6–8) of CT26 tumors on treatment with BUP-Gel, OX, and the combination of BUP-Gel with OX. (B–F) Change in the population (mean ± SEM, *n* = 4) of CD8^+^ (B), granzyme B^+^CD8^+^ and its MFI (C and D), TNF-α^+^CD8^+^ and its MFI (E and F) in untreated, BUP-Gel-treated, OX-treated, and CT26 tumors treated with the combination of BUP-Gel and OX. (G and H) Flow cytometry plots (G) and the percentage change (H) in CD8^+^ populations (mean ± SEM, *n* = 4) co-expressing granzyme B, IFN-γ, and TNF-α. (I) Tumor growth kinetics and final day tumor volume (mean ± SEM, *n* = 6) of B16 tumors on treatment with BUP-Gel, aCTLA4, and a combination of BUP-Gel with aCTLA4. (J–M) Change in the population (mean ± SEM, *n* = 4) of granzyme B^+^CD8^+^ (J), IFN-γ^+^CD8^+^ (K), IFN-γ^+^CD4^+^ (L), and TNF-α^+^CD4^+^(M) in untreated, aCTLA4-treated, BUP-Gel-treated, and combination of BUP-Gel and aCTLA4-treated B16 tumors. (N and O) Flow cytometry plots (N) and percentage change (O) in the CD8^+^ populations (mean ± SEM, *n* = 4) co-expressing granzyme B, IFN-γ, and TNF-α. (P) Tumor growth kinetics and final day tumor volume (mean ± SEM, *n* = 6–8) of B16-OVA tumors on treatment with BUP-Gel, OT-I T cells, and a combination of BUP-Gel with OT-I T cells. (Q–T) Change in the population (mean ± SEM, *n* = 4) of granzyme B^+^CD8^+^ and its MFI (Q and R), IFN-γ^+^CD8^+^ (S), MFI of TNF-α in CD8^+^(T) cells in untreated, OT-I T cell-treated, BUP-Gel-treated, and combination of BUP-Gel and OT-I T cell-treated B16-OVA tumors. (U and V) Flow cytometry plots (U) and percentage change (V) in the CD8^+^ populations (mean ± SEM, *n* = 4) co-expressing granzyme B, IFN-γ, and TNF-α. See also Figure S5 and Table S2. One-way ANOVA with Dunnett’s post-hoc test for (B)–(F), (H), (J)–(M), (O), (Q)–(T), and (V), and two-way ANOVA with Tukey’s post-hoc test for (A), (I), and (P). ∗*p* < 0.05, ∗∗*p* < 0.001, and ∗∗∗∗*p* < 0.0001 for the indicated comparisons.

As systemic chemotherapy and immunotherapy are the preferred treatment strategies for patients with cancer,^39^ we investigated whether BUP-Gel could improve the outcomes of clinically approved treatments. As oxaliplatin (OX) and 5-fluorouracil (5-FU) are first-line chemotherapeutics for patients with colon cancer,^40^ we tested the efficacy of systemic OX and 5-FU in combination with localized BUP-Gel therapy in CT26 tumor-bearing mice. OX therapy caused a ∼1.6-fold decrease in tumor growth, whereas the combination of BUP-Gel and OX resulted in ∼3.5- and ∼2-fold decreases in tumor growth compared with untreated and OX-treated tumors, yielding an additive effect (Figure 5A; Table S2). As OX is an inducer of immunogenic cell death (ICD),^41^ we analyzed the effect of the combination of BUP-Gel and OX on T cell activation. The combination of BUP-Gel and OX treatment led to a ∼1.1-fold increase in the frequency of infiltrated CD3^+^ T cells (Figure S5A) and a ∼2-fold increase in the frequency of CD8^+^ T cells compared to untreated tumors (Figure 5B). BUP-Gel and OX combination therapy also caused a ∼2-fold increase in granzyme B^+^CD8^+^ T cells with a >4-fold increase in their MFI (Figures 5C and 5D) and ∼3.5-fold increase in TNF-α^+^CD8^+^ T Cells with a ∼1.4-fold increase in its MFI compared to untreated tumors (Figures 5E and 5F). BUP-Gel and OX treatment also led to increased frequency of CD8^+^ T cells co-expressing granzyme B and TNF-α and co-expression of granzyme B, IFN-γ, and TNF-α compared to untreated tumors (Figures 5G and 5H). Further analysis of CD4^+^ T cells in these tumors revealed that BUP-Gel and OX treatment led to a ∼2-fold increase in granzyme B^+^CD4^+^ T cells with a ∼4-fold increase in its MFI, a >25-fold increase in IFNγ^+^CD4^+^ T Cells with >2-fold increase in its MFI and ∼9-fold increase in TNFα^+^CD4^+^ T cells, with a ∼1.5-fold increase in their MFI compared to untreated tumors (Figures S5B–S5G). We also observed a marked increase in the percentage of CD4^+^ T cells co-expressing IFN-γ and TNF-α, granzyme B and IFN-γ, granzyme B and TNF-α, and granzyme B, IFNγ, and TNF-α compared to untreated tumors (Figure S5H). Studying the effect of the combination of BUP-Gel and 5-FU revealed a ∼3.5-fold decrease in tumor growth compared to untreated tumors, whereas 5-FU caused only a ∼1.5-fold decrease (Figure S5I). As 5-FU is also an ICD inducer,^42^ the combination of BUP-Gel and 5-FU caused ∼1.3-fold increase in granzyme B^+^CD8^+^ T cells with a ∼1.6-fold increase in MFI (Figure S5J), and a >2-fold increase in IFNγ^+^CD8^+^ T cells compared to untreated tumors (Figure S5K). Interestingly, we observed ∼2-fold increase in granzyme B^+^IFN-γ^+^ and TNF-α^+^IFN-γ^+^ cells on treatment with a combination of BUP-Gel and 5-FU (Figures S5L and S5M).

Immune checkpoint blockade (ICB) therapy, including anti-PD1/anti-PD-L1 and anti-CTLA4 (aCTLA4), has gained attention for cancer treatment despite its low response rates.^43^ As BUP-Gel therapy enhances the activation of T cells along with CTLA4 expression on T cells, we hypothesized that localized BUP-Gel treatment might enhance the efficacy of aCTLA4 therapy. The combination of BUP-Gel and aCTLA4 showed ∼1.8-fold reduction of tumor growth in comparison to aCTLA4-treated tumors and a >4-fold decrease in tumor growth when compared with untreated B16 tumors (Figure 5I). Flow cytometric quantification of T Cells showed a ∼3.5-fold increase in granzyme B^+^CD8^+^ T cells, a ∼1.5-fold increase in IFN-γ^+^CD8^+^ T Cells, a ∼3-fold increase in IFN-γ^+^CD4^+^ T Cells and a ∼1.7-fold increase in TNF-α^+^CD4^+^ T cells in tumors treated with the combination of BUP-Gel and anti-CTLA4 compared to untreated tumors (Figures 5J–5M). Interestingly, treatment with BUP-Gel and anti-CTLA4 also led to increased frequency of CD8^+^ T cells co-expressing IFN-γ and TNF-α as well as granzyme B and IFN-γ compared to untreated tumors (Figures 5N and 5O).

Adoptive T cell transfer (ACT) uses tumor-reactive T cells that are expanded *ex vivo* and reinfused into patients to boost T cell-mediated killing of cancer cells.^44^ We employed OT-I T cells, which are reactive toward OVA-peptide-expressing B16 cells, in combination with localized BUP-Gel therapy against the B16-OVA tumor model. The combination of BUP-Gel with OT-I T cells showed a ∼4-fold and ∼8-fold reduction of tumor growth in comparison to OT-I T cell transfer alone and untreated tumors (Figure 5P). Flow cytometry revealed a ∼1.6-fold increase in granzyme B^+^CD8^+^ T cells with a ∼1.3-fold increase in MFI, a ∼5-fold increase in IFN-γ^+^CD8^+^ T cells, and a ∼1.4-fold increase in MFI of TNFα in CD8^+^ T cells upon treatment with the combination of BUP-Gel and OT-I T cells (Figures 5Q–5T). We observed enhanced frequency of CD8^+^ T cells co-expressing IFN-γ and TNF-α, granzyme B and TNF-α as well as all co-expression of granzyme B, IFN-γ, and TNF-α in BUP-Gel and OT-I treated tumors (Figures 5U and 5V). Analysis of BUP-Gel therapy in combination with OX, aCTLA4, and ACT showed additive effects on tumor regression (Table S2), suggesting that the combination therapy acts through independent, non-redundant pathways. Overall, these results suggest that localized BUP-Gel therapy can be combined with existing chemotherapy and immunotherapy regimens to achieve better efficacy and clinical outcomes.

### Antagonizing specific ACh receptors improve outcomes of chemotherapy and ICB therapy

**Figure 6:**
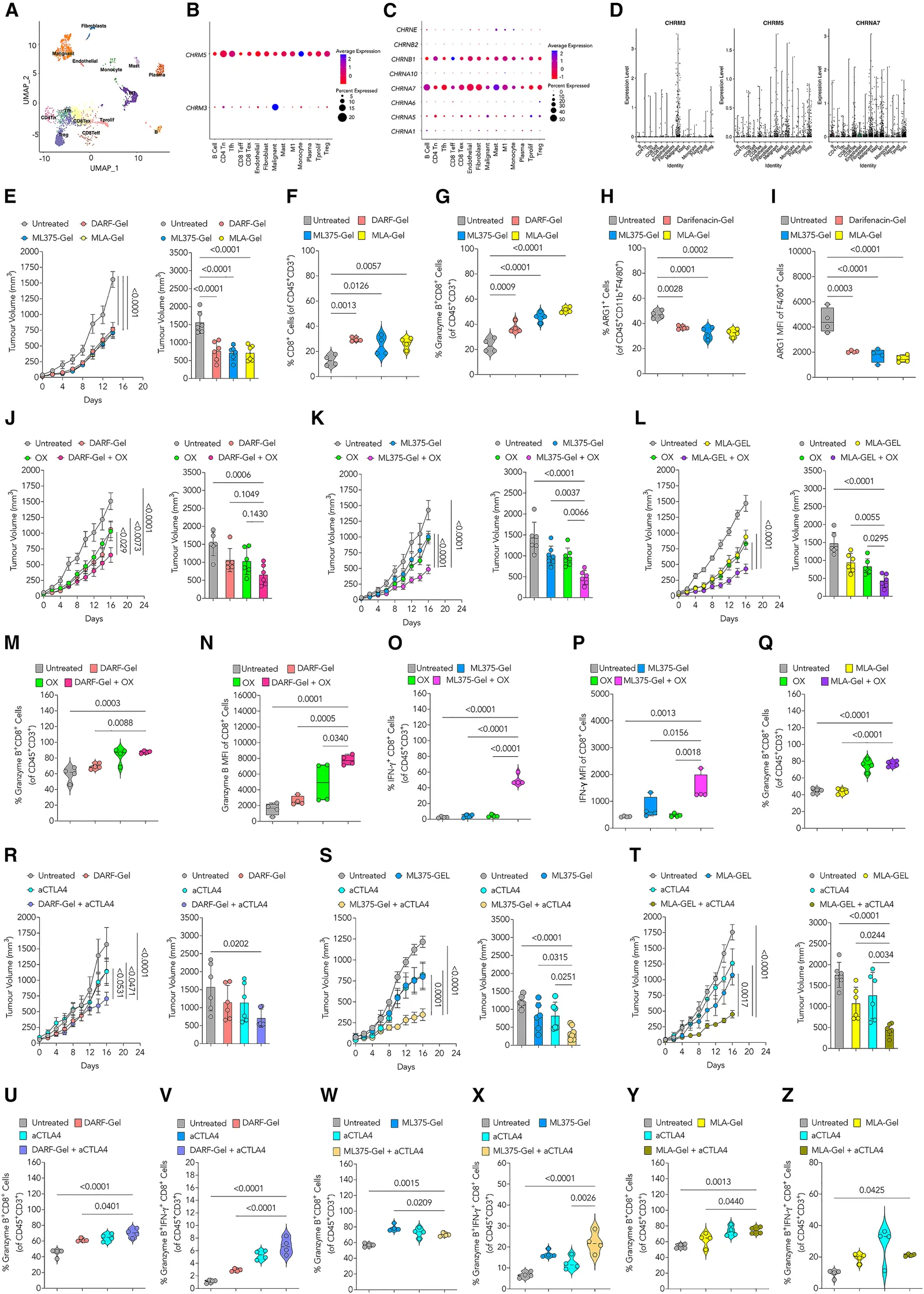
Antagonizing specific ACh receptors improve outcomes of chemotherapy and ICB therapy (A) *UMAP* plot showing different clusters after unsupervised clustering of a human scRNA sequencing dataset. (B and C) Dot plots represent the average expression (color) and the proportion of cells (dot size) expressing gene transcripts for muscarinic receptors (B) and nicotinic receptors (C) across different cell clusters. (D) Violin plot showing the expression of mAChRs, CHRM3, CHRM5, and nAChR, CHRNA7 across different cell clusters. (E) Tumor growth kinetics (mean ± SEM, *n* = 6) and final day tumor volume of CT26 tumors on treatment with DARF-Gel (M3 mAChR antagonist), ML375-Gel (M5 mAChR antagonist), and MLA-Gel (α7 nAChR antagonist). (F–I) Change in the population (mean ± SEM, *n* = 4) of CD8^+^ T cells (F), granzyme B^+^CD8^+^ (G), ARG1^+^ macrophages (H) and ARG1 MFI (I) within macrophages in tumors treated with DARF-Gel, ML375-Gel, and MLA-Gel. (J–L) Tumor growth kinetics and final day tumor volume (mean ± SEM, *n* = 6) of CT26 tumors on treatment with different receptor antagonists, OX, and their combinations. (M–Q) Change in the population (mean ± SEM, *n* = 4) of different immune cell types in CT26 tumors on treatment with different receptor antagonists, OX, and their combinations. (R–T) Tumor growth kinetics and final day tumor volume (mean ± SEM, *n* = 6) of CT26 tumors on treatment with different receptor antagonists, aCTLA4, and their combinations. (U–Z) Change in the population (mean ± SEM, *n* = 4) of different immune cell types in CT26 tumors on treatment with different receptor antagonists, aCTLA4, and their combinations. See also Figure S6 and Table S3. One-way ANOVA with Dunnett’s post-hoc test for (F)–(Z), and two-way ANOVA with Tukey’s post-hoc test for (E), (J)–(L), and (R)–(T). ∗*p* < 0.05, ∗∗*p* < 0.001, and ∗∗∗∗*p* < 0.0001 for the indicated comparisons.

ACh mediate signals through specific muscarinic and nicotinic ACh receptors (mAChRs and nAChRs), and these receptors are known to be expressed differentially on different cell types (Table S3).^45^ Therefore, we analyzed a publicly available colorectal cancer dataset and identified 15 distinct clusters using unsupervised clustering.^46^ We determined receptor expression across different clusters (Figures 6A–6D), and identified that malignant cells, macrophages, and T cells express mAChRs, *CHRM3*, and *CHRM5* (Figures 6B and 6D), whereas among nAChRs, *CHRNA7*, and *CHRNB1* are expressed by malignant cells, M1-like macrophages, and CD8^+^ T effector cells (Figures 6C and 6D). We next stratified patient tumors into high vs. low mAChR and nAChR expression and found that tumors with high mAChR expression exhibit increased Treg proportions and decreased M1-like macrophages (Figures S6A and S6B). In contrast, tumors with high nAChR expression were associated with reduced proportions of M1-like macrophages, accompanied by enrichment of exhausted CD8^+^ T cells (Figures S6C and S6D). These findings suggest that expression of these receptors may be linked to immunosuppressive cells in the TME.

Since immune cells and cancer cells expressed CHRM3, CHRM5, and CHRNA7 in the human scRNA dataset, we tested their protein expression in the CT26 tumor model and observed that these receptors are expressed in cancer cells, macrophages, and T cells within CT26 tumors (Figures S6E–S6G). We used specific antagonists to block these receptors and investigated the effect of hydrogel-mediated localized delivery of CHRM3 antagonist (darifenacin) (DARF-Gel), CHRM5 antagonist (ML375) (ML375-Gel), and CHRNA7 antagonist (methyllycaconitine citrate) (MLA-Gel) on CT26 tumor growth. Tumor growth kinetics revealed slower progression with all treatments, with >2-fold reductions in final-day tumor volume compared with untreated tumors (Figure 6E). Treatment of DARF-Gel, ML375-Gel, and MLA-Gel led to a >2-fold increase in the frequency of CD8^+^ T cells compared to untreated tumors (Figure 6F). We also observed a >1.5-fold increase in granzyme B^+^CD8^+^ T cells in tumors treated with these antagonists compared with untreated tumors (Figure 6G). Furthermore, we observed a ∼1.3-fold decrease in ARG1^+^ macrophages and a ∼2.5-fold decrease in ARG1 MFI in tumors treated with DARF-Gel, ML375-Gel, and MLA-Gel compared to untreated tumors (Figures 6H and 6I). To validate these findings, we assessed the effect of blocking ACh receptors on ACh-treated BMDM-derived M1-like macrophages (Figure S6H). Interestingly, antagonizing ACh receptors led to ∼1.2-fold increase in iNOS^+^ cells compared to ACh-treated M1 macrophages (Figure S6I). Similarly, all three antagonists showed >2-fold increase in IFN-γ^+^ cells compared to ACh-treated splenic CD8^+^ T cells (Figures S6J and S6K).

We next determined the efficacy of a combination of DARF-Gel, ML375-Gel, and MLA-Gel with systemic OX and aCTLA4 therapy in the CT26 tumor model and its impact on TME. A combination of DARF-Gel and OX resulted in slower tumor growth kinetics than other treatments, with a >2-fold decrease in tumor volume compared to untreated tumors (Figure 6J). We also observed a slower tumor growth rate in tumors treated with a combination of ML375-Gel and OX, with >3-fold decrease in tumor volume compared to untreated tumors and >2-fold decrease compared to OX-treated tumors (Figure 6K). Similarly, a combination of MLA-Gel and OX also hindered tumor growth, with ∼3-fold reduction compared to untreated tumors and ∼1.5-fold reduction compared to OX treatment (Figure 6L). Combination of DARF-Gel and OX caused a ∼1.5-fold increase in granzyme B^+^CD8^+^ cells with a ∼5-fold increase in granzyme B MFI compared to untreated tumors (Figures 6M and 6N). There was a ∼20-fold change in IFN-γ^+^CD8^+^ cells and a ∼3-fold change in its MFI upon treatment with a combination of ML375-Gel and OX compared to untreated tumors (Figures 6O and 6P). However, a combination of MLA-Gel and OX caused a >1.5-fold increase in granzyme B^+^CD8^+^ cells compared with untreated tumors (Figure 6Q), indicating that blocking ACh receptors enhance the efficacy of chemotherapy and improves T cell responses.

A combination of DARF-Gel and aCTLA4 resulted in diminished tumor kinetics with ∼2-fold and ∼1.5-fold reduction of tumor growth in comparison to untreated and aCTLA4-treated tumors (Figure 6R). Similarly, we observed ∼8-fold and ∼4-fold suppression of tumor growth in tumors treated with a combination of ML375-Gel and aCTLA4 compared to untreated and aCTLA4-treated tumors (Figure 6S). Use of aCTLA4 in combination with MLA-Gel also caused >2.5-fold decrease in tumor growth compared to aCTLA4-treated tumors (Figure 6T). Combination of DARF-Gel and aCTLA4 led to >1.5-fold increase in granzyme B^+^CD8^+^ and >5-fold increase in granzyme B^+^IFN-γ^+^CD8^+^ T cells compared to untreated tumors (Figures 6U and 6V). There was a ∼1.2-fold increase in granzyme B^+^CD8^+^ and >3.5-fold increase in granzyme B^+^IFN-γ^+^CD8^+^ T cells upon treatment with a combination of ML375-Gel and aCTLA4 (Figures 6W and 6X), along with ∼1.4-fold increase in granzyme B^+^CD8^+^ and >2-fold increase in granzyme B^+^IFN-γ^+^CD8^+^ T cells in tumors treated with MLA-Gel and aCTLA4 (Figures 6Y and 6Z). Analysis of targeting specific receptors in combination with OX and aCTLA4 showed additive effects on tumor regression (Table S2), suggesting that the combination therapy acts through independent, non-redundant pathways. As clinical treatments depend on cumulative effects for maximum benefits, combination therapeutic regimens are highly advantageous in clinical settings. Since chemotherapy and immunotherapy can cause toxicity, we evaluated liver and kidney parameters after treatment with different combinations and found no major changes in LFT (liver function test) and KFT (kidney function test) parameters (Figures S6L and S6M). These findings suggest that targeted antagonism of specific ACh receptors can suppress tumor growth without causing any systemic toxicity, reprogram the immune microenvironment, and enhance responses to conventional chemotherapy and immunotherapy. Collectively, these results indicate that while BUP-Gel directly impairs neuronal communication, blockade of cholinergic receptors with antagonists mediates parallel effects and represents a mechanistically distinct strategy.

### CHRNA7 antagonist mitigates ACh-mediated immunosuppression in patient-derived tumor explants

**Figure 7:**
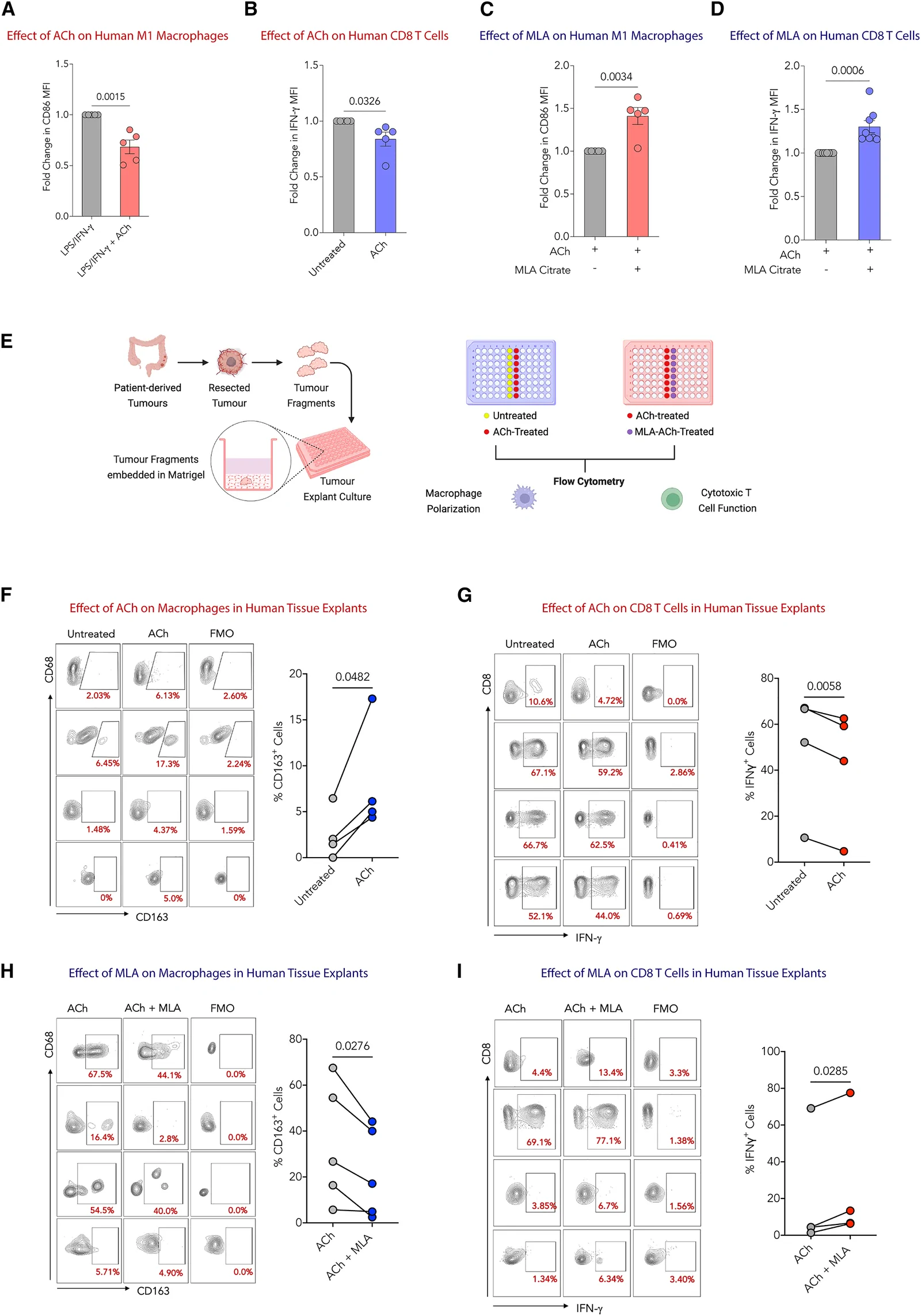
CHRNA7 antagonist mitigates immunosuppression in patient-derived tumor explants (A) Fold change in CD86 MFI (mean ± SEM, n = 5) in human monocyte-derived M1-like macrophages upon treatment with ACh. (B) Fold change in IFN-γ MFI (mean ± SEM, *n* = 5) in human CD8^+^ T cells upon treatment with ACh. (C) Fold change in CD86 MFI (mean ± SEM, *n* = 5) in human monocyte-derived M1-like macrophages treated with CHRNA7 antagonist MLA. (D) Fold change in IFN-γ MFI (mean ± SEM, *n* = 8) in human CD8^+^ T cells treated with CHRNA7 antagonist MLA. (E) A schematic showing the study design to decipher the impact of ACh and MLA on macrophages and T cells in patient-derived tumor fragments. (F) Flow cytometry plots and percentage change in CD163 expression in CD68^+^ cells (*n* = 4) in untreated and ACh-treated patient-derived tumor fragments from four different patients. (G) Flow cytometry plots and percentage change in IFN-γ expression in CD8^+^ cells (*n* = 4) in untreated and ACh-treated patient-derived tumor fragments from four different patients. (H) Flow cytometry plots and percentage change in CD163 expression in CD68^+^ cells (*n* = 5) in patient-derived tumor fragments upon treatment of MLA. (I) Flow cytometry plots and percentage change in IFN-γ expression in CD8^+^ cells (*n* = 4) in patient-derived tumor fragments upon treatment with MLA. See also Figure S7 and Table S2. Data among the two groups were analyzed using a two-tailed unpaired Student’s *t* test (A–D) and a paired Student’s *t* test (F–I). ∗*p* < 0.05, ∗∗*p* < 0.001, and ∗∗∗∗*p* < 0.0001 for the indicated comparisons.

As targeting ACh release using BUP-Gel or antagonizing ACh receptors using DARF-Gel, ML375-Gel, and MLA-Gel inhibited tumor growth and boosted immune surveillance in murine models, we determined the effect of ACh on human PBMC-derived macrophages and CD8^+^ T cells. We supplemented ACh to LPS/IFN-γ-induced M1-like macrophages (derived from human monocytes) and observed ∼1.5-fold reduction in CD86 expression (M1-marker) compared to untreated macrophages (Figure 7A; Figures S7A and S7B). We also assessed the effect of ACh on CD8^+^ T cells sorted from human PBMCs (Figure S7C). ACh treatment led to a ∼1.2-fold decrease in IFN-γ expression on CD8^+^ T cells compared with untreated CD8^+^ T cells (Figure 7B; Figure S7D), suggesting that ACh plays a key role in polarizing human macrophages and inhibiting the cytotoxic function of human T cells. Next, we analyzed the effect of one of the antagonists (MLA specific for CHRNA7) on human macrophages and T cells. Interestingly, we observed ∼1.4-fold increase in CD86 expression upon treatment of ACh-conditioned M1-like macrophages with MLA (Figure 7C; Figure S7E), and ∼1.3-fold increase in the IFN-γ expression of CD8^+^ T cells upon blocking CHRNA7 antagonist in ACh-treated human CD8^+^ T cells (Figure 7D; Figure S7F), thereby validating the CHRNA7-mediated effects of ACh in human immune cells.

Next, we cultured the patient-derived colon cancer tumor fragments in a collagen-based extracellular matrix and treated these tumor explants with ACh (Figure 7E).^47^^,^^48^ Interestingly, ACh treatment led to a >3-fold increase in CD163 expression (M2 Marker) on CD68^+^ macrophages compared to untreated colon cancer explants (Figure 7F; Figure S7G). ACh treatment also reduced the frequency of IFN-γ^+^ CD8^+^ T cells in the tumor-infiltrating CD8^+^ T cells compared to untreated patient tumors (Figure 7G; Figure S7H). Interestingly, treatment with CHRNA7 antagonist MLA diminished CD163 expression on macrophages in ACh-treated tumor fragments (Figure 7H) and increased the number of IFN-γ^+^ CD8^+^ T cells (Figure 7I). Therefore, these results validate our findings that blocking of CHRNA7-mediated signaling can abolish the ACh-mediated effects on macrophages and cytotoxic T cell functions, thereby stimulating anti-tumor immunity in colon cancer.

## Discussion

Tumors promote nerve innervation into the tumor niche by secreting neurotrophic factors, facilitating interactions among cancer cells, neurons, and immune cells via neurotransmitters and neuropeptides.^49^ This neuron-cancer-immune crosstalk modulates TME and contributes to tumor progression and immunosuppression.^50^ Previous studies demonstrated that the presence of NEFL- and PGP9.5-positive nerves within colon tumors is associated with poor prognosis and reduced overall survival in patients, as corroborated by our Kaplan-Meier survival analysis from publicly available databases.^51^^,^^52^ Moreover, multivariate Cox regression analysis also demonstrated independent prognostic significance of NEFL and ChAT in early stages, revealing probable involvement of cholinergic nerves in the progression of colon cancer independent of clinicopathological features, including age, laterality, and therapy. We further demonstrated that ∼47% of patients with colon cancer exhibit high levels of cholinergic neurons (NEFL and ChAT) in tumor tissues, and this is associated with reduced CD8^+^ T cell infiltration and increased macrophage accumulation. Furthermore, elevated ACh levels in tumor tissues compared with adjacent normal regions suggest active ACh-mediated communication within the TME.

ACh is one of the key neurotransmitters released from enteric nerves in the gastrointestinal tract, crucial for maintaining gut motility.^53^ Although non-neuronal cells, including cancer cells, can secrete ACh at nanomolar concentrations,^54^ these levels are modest and often regulated by local acetylcholinesterase activity.^55^ In contrast, neuronal ACh release occurs at substantially higher concentrations, ranging from 0.1 to 1 μM in the central nervous system to transient micromolar levels (10–100 μM) in peripheral nerves to mM concentrations at synaptic junctions.^56^^,^^57^ This highlights a greater potential of neuronal ACh to modulate TME through ACh signaling. To account for the rapid degradation of ACh, we utilised a higher ACh concentration than the intratumoral ACh concentrations detected by LC-MS/MS. Neuronal and tuft-cell-derived ACh promotes gastric tumorigenesis,^19^ while cholinergic innervation in prostate cancer promotes tumor growth, invasion, and metastasis.^58^ However, the effect of ACh is organ specific and concentration dependent, as cholinergic signaling has been shown to suppress cancer stem cells and liver metastatic growth in pancreatic cancer.^20^ Consistent with this, we demonstrated that ACh enhances cancer cell proliferation in both 2D and 3D culture models and modulates immune cells, suggesting engagement of neuronal ACh within the TME, thereby presenting a potential therapeutic target for local anesthetics.

A recent clinical trial revealed that peritumoral lidocaine (a local anesthetic) injection before surgery significantly improves disease-free and overall survival in patients with breast cancer.^28^ Although earlier studies focused on liposomal formulation of local anesthetics like BUP,^26^ systemic delivery of these drugs can be life threatening.^31^ Therefore, our study utilized a hydrogel-based localized system for slow and sustained release of BUP at the tumor that limits tumor growth more effectively than BUP solution, highlighting the benefit of localized hydrogel-based systems. Our A13-gel scaffold was shown to exhibit good biocompatibility, *in vivo* persistence with minimal immune infiltration, and gradual clearance over 21–50 days in rodents.^32^ However, further evaluation of long-term biodegradability, systemic safety, and manufacturing scalability is essential for clinical translation. While localized BUP-Gel treatment demonstrated immune activation in tumors, likely by inhibiting neuronal ACh release, BUP can also exert other pleiotropic effects, such as inducing autophagy in lung cancer cells, inducing apoptosis in rhabdomyosarcoma cells, and exerting anti-inflammatory effects in astrocytes.^27^^,^^59^^,^^60^ Moreover, as BUP induces broad neural blockade, the observed anti-tumor and immune regulatory effects may involve the influence of other neurotransmitter-mediated signals beyond cholinergic signals.

Cholinergic activation in macrophages via nAChRs signals suppresses pro-inflammatory responses, possibly through inhibition of NF-κB signaling and reduced TNF and IL-1 production.^61^ ACh released during perineural invasion in pancreatic cancer impairs CD8^+^ T cell infiltration, suppresses IFN-γ, and promotes a Th1-to-Th2 immune shift.^62^ In line with this, our results show that inhibiting ACh release using BUP-Gel reprograms macrophages toward a pro-inflammatory phenotype and enhances T cell cytotoxicity, which are abrogated by ACh supplementation. While these findings highlight the role of cholinergic signaling in immune suppression, the involvement of other neural inputs affected by BUP cannot be ruled out entirely.

Our scRNA-seq analysis in patients with colon cancer revealed predominant CHRM3 expression in malignant cells and macrophages, whereas CHRNA7 and CHRM5 were broadly expressed across immune cell populations. CHRM3 signaling has been demonstrated to support tumor growth and suppress anti-tumor immunity.^63^^,^^64^^,^^65^ Similarly, CHRNA7 signaling promotes tumor growth in gastric, colon, liver, and pancreatic cancers and suppresses macrophage pro-inflammatory cytokine production by downregulating NF-κB.^66^^,^^67^^,^^68^ Consistent with this, our results demonstrated tumor growth inhibition and improved anti-tumor immunity upon blockade of these receptors. In addition to modulating neuron-derived cholinergic signaling, therapeutic targeting of these receptors also reflects the direct effects on immune cells within the TME. Therefore, our findings suggest that while BUP-Gel primarily targets neural inputs to the TME by inhibiting ACh release, targeting of ACh receptors represent another parallel and distinct strategy to modulate the TME.

Therefore, in summary, hydrogel-mediated delivery of FDA-approved local anesthetics and cholinergic receptor antagonists can inhibit ACh-mediated tumor progression, reverse ACh-mediated immunosuppression, and augment responses to chemotherapy and immunotherapy.

### Limitations of the study

Although several studies have suggested a role for neurons in cancer progression, there is no evidence for the role of acetylcholine from cholinergic neurons in the colon TME. However, it is important to consider the following limitations while interpreting the results. First, the multivariate Cox regression analysis using the GSE1433 cohort did not adjust for MMR (mismatched repair) status, a crucial prognostic factor in colon cancer, due to the unavailability of data. Second, tumor harbors a mixed population of neurons, and BUP causes non-specific neuronal blockade, including cholinergic, sympathetic, sensory, and enteric neurons within the TME. While ACh supplementation and ACh receptor blockade indicates the role of cholinergic signaling in tumor growth and immunosuppression, the involvement of each neural input requires further investigation. Third, non-neuronal sources of ACh, including cancer cells, stromal cells, and tuft cells, also contribute to the TME, and their individual roles need to be elucidated. Fourth, replicating physiological ACh concentrations is challenging, as ACh levels vary across tissues and are rapidly degraded by acetylcholinesterase. Lastly, the response to cholinergic receptor blockade in our patient-derived tumor explant studies may be influenced by the baseline immune composition of individual patient tumors, a finding that requires further investigation to better define patient-specific responses. In addition, patient-derived tumor explant studies were limited to MLA treatment only and not performed with darifenacin or ML375 due to limited tissue availability.

## Resource availability

### Lead contact

Further information and requests for resources and reagents should be directed to and will be fulfilled by the lead contact, Avinash Bajaj (bajaj@rcb.res.in).

### Materials availability

This study did not generate unique reagents.

### Data and code availability

• The raw sequencing data in this study have been deposited in Gene Expression Omnibus (GEO: GSE303917). The remaining data are available within the article and supplemental information.
• This paper does not report original code.
• Any additional information required to reanalyze the data reported in this paper is available from the lead contact upon request.

## Acknowledgments

Tissue samples had been collected for research purposes with consent. Schematic figures were created using BioRender.com. We thank the biorepository of Rajiv Gandhi Cancer Institute and Research Center (RGCIRC), Delhi. The students thank DBT, ICMR, UGC, and CSIR for research fellowships. We thank Dr. Sunil Raghav and Dr. Shilpak Chatterjee for their helpful discussions and suggestions. We acknowledge the support of the DBT e-Library Consortium (DeLCON) for providing access to e-resources. Research in the AB group is supported by ANRF, 10.13039/501100001407DBT, 10.13039/501100001411ICMR, and 10.13039/100017220RCB Core Funding. Animal work in the small animal facility of the Regional Center for Biotechnology is supported by the Department of Biotechnology.

## Author contributions

A.B. conceived the study, acquired funding, administered and supervised the project, and provided resources. D.J., N.P., S.K.J., S.S., R.S., J.A., B.A., A.K., N.K.C., K.R., P.Y., A.K.S., D.N., J.R., and J.S.P. curated the data. D.J., N.P., S.K.J., S.S., R.S., A.A., and A.K. performed formal data analysis. D.J., N.P., S.K.J., S.S., R.S., A.P.V., A.K., L.M., S.R., S.K.B., R.P. A.P., A.M., V.D., R.B., and J.S.P. carried out the investigation. D.J., N.P., S.K.J., A.K., B.A., N.C., A.P.V., S.K., and U.D. developed the methodology. V.D., R.B., P.D., V.S.P., U.D., and A.B. provided resources. U.D., S.S., J.S.P., V.S.P., and A.B. supervised the study. D.J., N.P., and S.K.J. validated the findings. D.J., P.D., J.S.P., and V.S.P visualized the data. D.J. and A.B. wrote the manuscript, reviewed, and edited the final version.

## Declaration of interests

The authors declare that they have no competing interests.

## STAR★Methods

### Key resources table

**Table:** 

REAGENT or RESOURCE	SOURCE	IDENTIFIER
**Antibodies**		

Anti-Arginase 1 (A1exF5) PE-Cy7	ThermoFisher	Cat# 25-3697-82; RRID:AB_2734841
Anti-CHAT Human	ProteinTech	Cat# 20747-1-AP; RRID:AB_10898169
Anti-NF-L Human	ProteinTech	Cat# 60189-1-Ig; RRID: AB_10860271
Anti-CD68 Human	ProteinTech	Cat# 66231-2-Ig; RRID: AB_2881622
Anti-CD8a	ProteinTech	Cat# 66868-1-Ig; RRID: 2882205
Anti-CHRM3 Human	ThermoFisher	Cat# MA5-38381; RRID: AB_2898295
Anti-CHRM5 Human	ThermoFisher	Cat# PA5-106925; RRID: AB_2854589
Anti-CHRNA7 Human	ThermoFisher	Cat#ANC-007; RRID: AB_10659339
Anti-CD68 Antibody	ThermoFisher	Cat#PA5-143237; RRID: AB_2933876
Anti-CD8a	ThermoFisher	Cat#740029T; RRID:AB_3679132
Anti-mouse CD45-FITC	BioLegend	Cat# 103108; RRID:AB_312973
Anti-mouse iNOS-PE	BioLegend	Cat# 696806; RRID:AB_2876745
Anti-mouse Ly6G-percp/Cy5.5	BioLegend	Cat# 127616; RRID:AB_1877271
Anti-mouse F4/80-APC	BioLegend	Cat# 157306; RRID:AB_2832549
Anti-mouse/human CD11b-APC/Cy7	BioLegend	Cat# 101226; RRID: AB_830642.
Anti-mouse CD8a-FITC	BioLegend	Cat# 100706; RRID:AB_312745
Anti-mouse/human Granzyme B-PE	BioLegend	Cat# 396406; RRID:AB_2801075
Anti-Granzyme B-PE/Dazzle 594	BioLegend	Cat# 372216; RRID:AB_2728383
Anti-mouse CD45-percp/Cy5.5	BioLegend	Cat# 103132; RRID:AB_893340
Anti-mouse CD3-BV421	BioLegend	Cat# 100336; RRID: AB_11203705
Anti-mouse CD4-PE/Cy7	BioLegend	Cat# 100422; RRID:AB_312707
Anti-mouse IFN-γ-AF700	BioLegend	Cat# 505824; RRID:AB_2561300
Anti-mouse TNF-α-BV785	BioLegend	Cat# 506341; RRID:AB_2565951
Anti-mouse CD152-PE	BioLegend	Cat# 106306; RRID:AB_313255
Anti-mouse TIM-3-APC	BioLegend	Cat# 134008; RRID:AB_2562998
Anti-mouse CD279-APC/Cy7	BioLegend	Cat# 135224; RRID:AB_2563523
Anti-mouse CD3-PE/Cy7	BioLegend	Cat# 100320; RRID: AB_312685
Anti-mouse CD4-APC/Cy7	BioLegend	Cat# 100414; RRID: AB_312699
Anti-mouse CD44-PE	BioLegend	Cat#103008; RRID: AB_312959
Anti-mouse CD62L-PerCP/Cy5.5	BioLegend	Cat#104432; RRID: AB_2285839
Anti-human CD45-AF700	BioLegend	Cat# 368514; RRID:AB_2566374
Anti-human CD3e-PE/Cy7	BioLegend	Cat# 300420; RRID:AB_439781
Anti-human CD8a-FITC	BioLegend	Cat# 301006; RRID:AB_314124
Anti-human CD86-FITC	BioLegend	Cat# 374204; RRID:AB_2721574
Anti-human CD68-PE/Cy7	BioLegend	Cat# 333816; RRID:AB_2562936
Anti-human CD163-PE	BioLegend	Cat# 326506; RRID:AB_893265
Anti-human IFN-γ-APC	BioLegend	Cat# 502512; RRID:AB_315237
anti-mouse CD152 Antibody	BioLegend	Cat# 106214; RRID: AB_2813939
anti-mouse CD4 Antibody	BioLegend	Cat# 100458; RRID:AB_11149488
anti-mouse CD8a Antibody	BioLegend	Cat# 100764; RRID:AB_2810324
IgG2b Isotype Control	BioLegend	Cat# 400672; RRID: AB_11149687
Anti-CD68 Human	Invitrogen	Cat# PA5-143237; RRID: AB_2933876
Biotin anti-mouse CD8a	Biolegend	Cat# 100703-50μg; RRID: AB_312742
Alexa Fluor® 488 Goat Anti-Mouse IgG	Jackson ImmunoResearch	Cat#115-547-003; RRID: AB_2338869
Alexa Fluor® 594 Goat Anti-Mouse IgG	Jackson ImmunoResearch	Cat#115-586-003; RRID: AB_2338890
Alexa Fluor® 647 Donkey Anti-Goat IgG	Jackson ImmunoResearch	Cat#705-607-003; RRID: AB_2340439
Alexa Fluor® 488 Donkey Anti-Goat IgG	Jackson ImmunoResearch	Cat#705-547-003; RRID: AB_2340431
Alexa Fluor® 488 Streptavidin	Jackson ImmunoResearch	Cat#016-540-084; RRID: AB_2337249
Alexa Fluor® 647 Streptavidin	Jackson ImmunoResearch	Cat#016-600-084; RRID: AB_2341101

**Biological samples**		

Human PBMCs	This Paper	N/A
Human Colon cancer Tissue Samples	This Paper	N/A

**Chemicals, peptides, and recombinant proteins**		

RPMI-1640	Sigma-Aldrich	Cat# R6504
DMEM	Sigma-Aldrich	Cat# D5648
DPBS	Sigma-Aldrich	Cat# 56064C
Fetal Bovine Serum	ThermoFisher	Cat# A5256701
Antibiotic-Antimycotic	ThermoFisher	Cat#15240062
Phorbol 12-myristate 13-acetate	Sigma-Aldrich	Cat# 79346-5MG
Collagenase type IV	Sigma-Aldrich	Cat# C5138-5G
DNase I from bovine pancreas	Sigma-Aldrich	Cat# D4527-200KU
Bupivacaine	Sigma-Aldrich	Cat# B5274-5G
Percoll	Sigma-Aldrich	Cat# 17089101
DPX mount	Sigma-Aldrich	Cat# 06522
Xylene	Sigma-Aldrich	Cat# 534056
TWEEN® 20	Sigma-Aldrich	Cat# P7949
Lipopolysaccharides (E. coli O111:B4)	Sigma-Aldrich	Cat# L4130
DAB	Sigma-Aldrich	Cat# D3939
Matrigel® Basement Membrane Matrix	Corning	Cat# 354234
Carbamoylcholine chloride	Merck	Cat# C4382-1G
Ionomycin	Merck	Cat# I0634-1MG
Ethanol	Merck	Cat# 100983
Human Serum	Merck	Cat# H4522
Collagen I rat tail	ThermoFisher	Cat# A1048301
Gentamicin	ThermoFisher	Cat# 15750060
MEM Non-Essential Amino Acids Solution (100X)	ThermoFisher	Cat# 11140050
Iscove’s Modified Dulbecco’s Medium	Hyclone	Cat# SH30228.01
Hi-AR™	HiMedia	Cat# RM1131-1G
Bovine serum albumin fraction-V	HiMedia	Cat# MB083-100G
Sodium bicarbonate	HiMedia	Cat# TC230
Goat serum	HiMedia	Cat# RM10701
Hisep-LSM 1077	HiMedia	Cat# LS001
Sodium pyruvate	HiMedia	Cat# TCL015
2-Mercaptoethanol	HiMedia	Cat# MB041
10X RBC Lysis Buffer	HiMedia	Cat# R075
L-Glutamine Solution	HiMedia	Cat# TCL012
iScript cDNA synthesis kit	Bio-Rad	Cat# 1708891
iTaq universal SYBR Green supermix	Bio-Rad	Cat# 1725124
RNAiso Plus	DSS Takara	Cat# 9109
Disodium hydrogen orthophosphate dihydrate	SD Fine Chemicals	Cat# 40158
Sodium hydroxide	SD Fine	Cat# 40167
Darifenacin hydrobromide	MedChemExpress	Cat# HY-A0012
ML375	MedChemExpress	Cat# HY-12567
Methyllcaconitine citrate	MedChemExpress	Cat# HY-N2332A
MS-grade methanol	Honeywell	Cat# 34966
Acetonitrile	Honeywell	Cat# 34967
Water	Honeywell	Cat# 39253-4L
Isopropanol	Honeywell	Cat# 34965
Ammonium acetate	Honeywell	Cat# 14267
Acetylcholine-d9	MedChemExpress	Cat# HY-B0282S1
Acetylcholine	MedChemExpress	Cat# HY-B0282
Choline	Sigma-Aldrich	Cat# 67-48-1
Acetylcholine	Sigma-Aldrich	Cat# A2661-25G
Dacotin (Oxaliplatin)	Dr. Reddy’s	NA
5-Flucel (5-FU)	Celon Labs	NA
Monensin Solution (1,000X)	BioLegend	Cat# 420701
Zombie Aqua Fixable Viability Kit	BioLegend	Cat# 423102
Recombinant Mouse IFN-γ	BioLegend	Cat# 575306
Cyto-Fast™ Fix/Perm Buffer Set	BioLegend	Cat# 426803
Recombinant Human IFN-γ	BioLegend	Cat# 570206
Recombinant Mouse IL-2	BioLegend	Cat# 575404
Recombinant Human M-CSF	BioLegend	Cat# 574804
Recombinant Human IL-2	BioLegend	Cat# 791904
Zombie Aqua Fixable Viability Kit	BioLegend	Cat# 423102
Dynabeads™ Mouse CD3/CD28	ThermoFisher	Cat# 11452D
Dynabeads™ Human CD3/CD28	ThermoFisher	Cat# 11131D
Pierce™ BCA protein assay kit	ThermoFisher	Cat# 23227
UltraComp Compensation Beads	ThermoFisher	Cat# 01-2222-41

**Critical commercial assays**		

Human CD14^+^ Isolation kit	BioLegend	Cat# 480048
Ultratek HRP Anti Polyvalent Staining S	Scytek	Cat# AFN600
Ultratek HRP Anti Rabbit Red Stain	Scytek	Cat# AME080

**Deposited data**		

scRNA	NCBI	GSE303917

**Experimental models: Cell lines**		

CT26	ATCC	Cat.# ATCC CRL 2638; RRID:CVCL_7256
4T1	ATCC	Cat.# ATCC CRL-2539; RRID: CVCL_0125
B16	ATCC	Cat#CRL-6475; RRID:CVCL_0159
B16-OVA	This Paper	NA
N2A	NCCS	NA

**Experimental models: Organisms/strains**		

C57/BL6J	The Jackson Laboratory	Cat#/Strain #:000664; RRID:IMSR_JAX:000664
BALB/cJ	The Jackson Laboratory	Cat# /Strain #:000651; RRID:IMSR_JAX:000651
C57BL/6-Tg(TcraTcrb)1100Mjb/J	Jackson Laboratory	Cat# /Strain #:003831; RRID:IMSR_JAX:003831
NOD.Cg-Prkdcscid Il2rgtm1Wjl/SzJ	The Jackson Laboratory	Cat# /Strain# 005557; RRID:IMSR_JAX:005557

**Oligonucleotides**		

Primers for RT-qPCR	This paper	See Table S1 for details

**Software and algorithms**		

GraphPad Prism v10.6.1	GraphPad Inc.	RRID:SCR_002798
FlowJo v10.1	BD Biosciences	RRID:SCR_008520
Seurat v5.3.0	Satija Lab	RRID:SCR_016341
RStudio	Posit PBC	RRID: SCR_000432

### Experimental model and study participant details

#### Patient samples

All studies with human samples were conducted after due ethical clearance from Institute Ethics Committee (Human) of All India Institute of Medical Sciences New Delhi (Approval No. AIMSA2295/07.10.2024, RP-43/2024), Institute Review Board of Rajiv Gandhi Cancer Institute and Research (Approval No. BR-2017- 007), Institute Ethics Committee (Human) of ESIC Medical College and Hospital Faridabad (Approval No. 134 X/11/13/2025-IEC/DHR/04) and Institute Ethics Committee (Human) of Regional Center for Biotechnology (Approval No. RCB-IEC-H-47, RCB-IEC-H-52) after the due written consent from the patients.

Colon cancer tissues from patients undergoing surgery were collected from the All India Institute of Medical Sciences, New Delhi, India, and from the biorepository of Rajiv Gandhi Cancer Institute and Research Center (RGCIRC), Delhi, India. Inclusion criteria for patients with colon cancer include those at any stage undergoing upfront surgery. Clinical details of the patients are provided in DataSet 1. Blood samples of healthy individuals were obtained from ESIC Medical College and Hospital, Faridabad, India. Inclusion criteria for blood from healthy individuals include being clinically healthy, without any chronic or acute illnesses, and having no history of significant medical conditions such as cardiovascular, renal, or infectious diseases.

All tissue specimens obtained as primary surgical resections were processed according to the center’s standard operating procedures. Samples were transferred to the department of pathology immediately after resection, where representative tumor sections were fixed in 10% neutral buffered formalin within the recommended time. The fixation duration, paraffin embedding, and sectioning were performed according to institutional QC guidelines to ensure optimal preservation of morphology and antigenicity. Only samples that met predefined quality criteria, including adequate tumor cellularity with preserved tissue architecture representative of the tumor, were included in this study. For patient-derived tumor explant studies, samples were immediately collected in cold sterile transport medium (RPMI supplemented with 10% FBS and 1% penicillin/streptomycin) and kept on ice. Samples were processed within 1–2h of surgical resection to minimize tissue degradation.

We performed a post hoc power analysis for categorical comparisons (high vs. low cholinergic neuron density correlated with CD8^+^ and CD68^+^ immune cell infiltration). Assuming a medium-to-large effect size (Cohen’s w = 0.4) and α = 0.05, the expanded cohort (*n* = 68) provides approximately 90–95% power for detecting associations with CD68^+^ macrophage infiltration, indicating robust statistical sensitivity for this comparison. However, for CD8^+^ T cell infiltration, the effect size was smaller (Cohen’s h ∼0.18), suggesting a small to moderate association, and a larger cohort size will be needed to validate the trend.

#### Animal studies

All the animal experiments were approved by the Institute Animal Ethical Committee of the Regional Center for Biotechnology (Approval no. RCB/IAEC/2022/133, RCB/IAEC/2023/166, RCB/IAEC/2023/237, RCB/IAEC/2024/215) and the National Institute of Immunology (Approval no. IAEC#680/24). We used 6-8-week-old BALB/c male mice or NSG male mice for experiments involving CT26 cells, 6-8-week-old female BALB/c mice for experiments involving 4T1 cells, and 6-8 week-old male C57/BL6 mice for experiments pertaining to B16 and B16-OVA cells.

#### Cell lines

4T1 and CT26 were cultured in RPMI-1640 containing 10% FBS (Gibco, Carlsbad, CA, USA) and 1% Penicillin-streptomycin solution (Gibco, Carlsbad, CA, USA). N2A, L929, B16 and B16-OVA cell lines were cultured in DMEM (Sigma-Aldrich, St. Louis, USA) containing 10% FBS (Gibco, Carlsbad, CA, USA) and 1% Penicillin-streptomycin solution (Gibco, Carlsbad, CA, USA). Cells were cultured in a humidified incubator at 37°C with 5% CO_2_ and confirmed to be free of mycoplasma.

### Method details

#### Cell culture studies

4T1, CT26, and N2A cells were seeded at 5,000 cells/well (96-well plate). After 24 h, cells were treated with serially diluted BUP (starting at 500 μM) and incubated for 48h. Viability was quantified using MTT at 570 nm (SpectraMax i3X, Molecular Devices, San Jose, USA).

To collect conditioned media, N2A and CT26 cells were grown in T75 flasks until 70–80% confluency and left untreated (CM (UT)) or treated with BUP (100 μM) (CM(BUP)). After 24h, conditioned media were collected for downstream experiments.

For the proliferation assay, 4T1, CT26, and N2A cells were seeded at 2,000 cells/well. After 24 h, cells were treated with ACh (50 μM), carbachol (50 μM), BUP (100 μM), or BUP + ACh for 24, 48, and 72h. For conditioned-media experiments, 4T1 and CT26 cells were treated with 50% CM (UT), CM (BUP), or CM (BUP) supplemented with ACh (50 μM) or carbachol (50 μM) for the same time points. Proliferation was measured by MTT at 570 nm.

For the spheroid assay, spheroids (4T1 or CT26 cells) were generated using the hanging-drop method.^69^ Drops (10 μL with ∼1,000 cells) were placed on Petri dish lids and incubated as untreated or with ACh (50 μM) or carbachol (50 μM) for 72 h. Co-spheroids (CT26/4T1: N2A = 9:1; 900 + 100 cells) were prepared and treated with BUP (100 μM) with/without ACh (50 μM) for 72h. Spheroids were imaged using Nikon Eclipse Ts2 (Nikon, Tokyo, Japan), and average diameters were quantified.

#### Immunohistochemistry

FFPE tissue sections (∼5 μM) were deparaffinized and rehydrated through a series of xylene, graded ethanol to distilled water washes. Tissue sections were then subjected to heat-induced epitope retrieval using citrate buffer (pH 6.0) for 30 min. Sections were then blocked for endogenous peroxidase activity with 3% hydrogen peroxide for 10 min, followed by protein blocking for 30 min. Primary antibodies, including anti-CD8α (ProteinTech, 1:2000) and anti-CD68 (ProteinTech, 1:1000), were used and incubated overnight at 4°C in a moist chamber. Slides were then incubated with Ultratek HRP Anti Polyvalent Staining System (Scytek), and detection was performed using 3,3′-diaminobenzidine (DAB) until a brown precipitate formed. For dual staining of NEFL and ChAT, slides were stained with anti-NEFL (ProteinTech, 1:200) raised in mouse as described above, followed by DAB treatment. Slides were then rinsed, reblocked and incubated with anti-ChAT antibody (ProteinTech, 1:100) raised in rabbit overnight at 4°C (pH 6.0) in a moist chamber. This was followed by incubation and detection with Ultratek ALP Anti Rabbit Permanent Red Stain (Scytek). All sections were then counterstained lightly with hematoxylin, rinsed in water, and mounted. Staining was evaluated by brightfield microscopy using an Olympus ch20i microscope (Olympus, Tokyo, Japan).

NEFL/ChAT expression in tumor sections (*n* = 68) was quantified by a certified pathologist. Each tumor tissue was examined in the tumor-dense and adjoining areas to determine the number of NEFL/CHAT-positive neurons per high-power field (HPF) and to derive a quantitative score. Tumors were then stratified into high and low groups based on the median value as the cut-off. For CD68^+^ macrophage and CD8^+^ T cell quantification, the numbers of CD68^+^ cells and CD8^+^ cells were counted in multiple (at least 5) HPF. CD8^+^ T cells were further stratified into high and low based on median cut-offs. The average number of infiltrating cells per HPF was determined for each tumor and used for statistical analysis. Detailed scoring has been provided in DataSet 2.

#### Multiplex immunofluorescence imaging

For immunofluorescence staining, 10 μm Formalin-Fixed Paraffin-embedded (FFPE) tissue sections were cut using a microtome (Leica RM2265). The sections were transferred to frosted slides (VWR) and dried overnight. Sections were deparaffinized using xylene and rehydrated with a series of ethanol to distilled water. Heat-induced epitope retrieval was performed by boiling the tissue sections in citrate buffer (pH 6.0) for 30 min. The sections were then washed and immuno-labeled using antigen-specific primary antibodies: anti-NEFL (ProteinTech), anti-CD68 (Invitrogen) and biotin-conjugated mouse anti-CD8a (BioLegend), followed by fluorophore-conjugated secondary antibodies or fluorescently conjugated streptavidin (Jackson ImmunoResearch Laboratories Inc.). Hoechst 33342 (Sigma Aldrich) was used to counterstain the nuclei. Sections were mounted using Prolong Gold antifade mounting medium (Invitrogen). Fluorescence imaging was performed using Leica STELLARIS 5 confocal microscope. Images were captured using 20X and 40X objectives and processed using LasX software^70^

#### Acetylcholine quantification using LC-MS/MS and data analysis

The cell pellets or tissues were resuspended in MS-grade water and homogenized by bead beating and probe sonication. An aliquot was taken for protein estimation using the Bicinchoninic acid (BCA) protein estimation kit. The cell suspension was incubated overnight at 4°C in 500 μL of 80% MeOH containing the acetylcholine-d9 internal standard, then centrifuged.^71^ The supernatant was transferred to another tube, and the pellet was extracted twice and dried. Dried samples were resuspended in 200 μL of 80% MeOH. The samples were vortexed, centrifuged, and transferred to the autoinjector vial for LC-MS/MS analysis. Analysis was performed using UHPLC (Exion LC AC, SCIEX, USA) coupled to a hybrid triple quadrupole/linear ion trap mass spectrometer (QTRAP 6500+, SCIEX, USA) using multiple reaction monitoring (MRM).^72^ The limits of detection, accuracy, precision, and matrix effect were not evaluated separately. For chromatographic separation, an AQUITY UPLC BEH X Bridge amide column (Waters) (3.5 μm, 4.6 × 150 mm) was used with an oven temperature of 35°C. Total run time was 18 min, where solvent A (95% acetonitrile with 10 mM ammonium acetate) and solvent B (50% acetonitrile with 10 mM ammonium acetate) were used as mobile phase A and B, respectively. The LC gradient solvent followed an increase in solvent B from 0% to 6% in 6 min, 25% for the next 4 min, 98% in the next 1 min, and 100% in the next 2.5 min with a flow rate of 0.7 mL/min. Finally, 100% buffer B was maintained for another 0.5 min at a flow rate of 1.5mL/min. Buffer B was then returned to 0% for 0.2 min, followed by equilibration for 3.8 min. Acquisition was performed using Analyst 1.6.3 software, and peak areas for all analytes were integrated using Multiquant 3.0.2 software (AB SCIEX, USA) for data analysis.

#### Hydrogel preparation and characterization

For BUP-Gel, 4 mg BUP was dissolved in 1 mL filtered Milli-Q water and added to 70 mg A13 hydrogelator. BUP-ACh-Gel and BUP-CARB-Gel were similarly prepared by incorporating ACh or carbachol at 0.5 mg/mL along with 4 mg BUP. The mixtures were heated, sonicated, and allowed to cool for gelation. The warm drug-loaded solution was transferred to a syringe, cooled to form the gel, and subsequently used for animal studies via a 22–26 gauge needle.^73^

Mechanical properties were measured using a parallel-plate rheometer (Anton Paar MCR302, Graz, Austria, RheoCompass 1.31; PP12.5 geometry, 0.5 mm gap). The linear viscoelastic region was first determined by performing an amplitude sweep (0.1–500% strain) at a constant angular frequency of 10 rad s^−1^. Based on this, an oscillatory frequency sweep (0.1–200 rad s^−1^) was conducted at 0.1% strain to evaluate the storage modulus (G′) and loss modulus (G″). Temperature-dependent rheological behavior was assessed via a temperature sweep from 20°C to 60°C at a heating rate of 2°C min^−1^, maintaining a constant oscillatory strain of 1% and an angular frequency of 6.28 rad s^-1^. To investigate the self-healing properties of the hydrogel network, thixotropic measurements were performed. The gel structure was initially disrupted by applying a high strain of 100%, followed by a reduction to 0.1% strain to monitor recovery. Additionally, three consecutive cycles of alternating high strain (100%) and low strain (0.1%) were applied at a constant angular frequency of 10 rad s^−1^ to evaluate the reversible breakdown and recovery behavior of the hydrogel.

For AFM imaging, 6 μL of sample was drop-cast onto a silicon wafer, incubated for 5 min, washed with 100 μL water, and air-dried in a desiccator. Height images were obtained in tapping mode using an Asylum Research MFP-3D (Santa Barbara, California, USA) with a silicon cantilever (Budget Sensor). Surface roughness was analyzed using IgorPro software.

For drug release studies, a 200 μL gel sample containing BUP was placed in a transwell with 500 μL release medium (1× PBS, pH 7.4, with 0.05% Tween 20) and incubated at 37°C. At predetermined time points, 500 μL of the medium was collected and replaced with fresh medium. Samples were speed-vacuum concentrated, methanol-extracted, and analyzed by HPLC (Waters, C18 column 4.6 × 250 mm, 5 μm; UV detection at 254 nm). Elution was performed using 50% acetonitrile and 50% solvent mixture [water (37.5): methanol (60): tetrahydrofuran (2.5) with 0.1% aq. NH_3_] at 0.5 mL/min.

#### Studies with mouse macrophages

Murine bone marrow cells were isolated from C57BL/6 mice. Briefly, the femur and tibia were dissected, and connective tissue was removed. Each bone cavity was flushed with DMEM high glucose supplemented with 10% FBS. Cells were collected in a 15 mL tube and centrifuged at 500g for 5 min. Pellet was then resuspended in 1mL of 1X RBC lysis buffer and incubated for 5 min. Falcon tubes (15 mL) were then filled with DPBS and passed through a 40 μm strainer to remove clumps and debris. Cells were spun at 500g for 5 min, and the supernatant was discarded. Cell pellet was resuspended in 20% L929 conditioned medium in complete DMEM and seeded in a 12-well plate or T-75 flask. After 6–7 days, bone marrow cells were differentiated into BMDMs and were used for further experiments.

For gene expression, BMDMs were either left untreated or treated with BUP (100 μM) or with 50% conditioned media from untreated N2A cells or BUP-treated N2A cells, incubated for 48h, and later processed for RNA isolation. For flow cytometry, BMDMs were polarized into M1-like BMDMs using LPS (100 ng/mL) and IFN-γ (20 ng/mL) for 24h. LPS/IFN-γ were removed, and M1-like BMDMs were subjected to different treatments for 48 h and processed for flow cytometry.

#### Studies with human macrophages

For human macrophages, CD14^+^ monocytes were sorted from the human peripheral blood mononuclear cells (PBMCs) of healthy individuals using MojoSort Human CD14^+^ Monocytes Isolation Kit as per the manufacturer’s protocol. Monocytes were cultured in complete RPMI-1640 supplemented with 50 ng/mL M-CSF. Media was refreshed every 2–3 days for 7–8 days. Monocyte-derived macrophages were polarized into M1-like macrophages using LPS (100 ng/mL) and IFN-γ (20 ng/mL) for 24h. LPS/IFN-γ were removed, and M1-like macrophages were subjected to different treatments for 48h and processed for flow cytometry.

#### Studies with mouse T cells

For murine T cells, the spleen from C57BL/6 mice was homogenized in ice-cold 1X PBS using frosted glass slides. The cell suspension was filtered through a 40 μm strainer, centrifuged at 1800 rpm for 5 min, and treated with 1X RBC lysis buffer for 4–5 min at room temperature. After washing with PBS and centrifugation, the pellet was resuspended in a 0.6 mL Magnetic-activated Cell Sorting (MACS) buffer, and cells were counted. Anti-mouse CD8 magnetic beads (100 μL per 10^7^ cells) were added and incubated at 4°C for 45–60 min. Following magnetic separation and two washes with MACS buffer, cells were resuspended in 1 mL T cell medium (RPMI with essential amino acids and glutamine) and seeded in a 48-well plate. These cells were stimulated with anti-CD3/CD28 magnetic dynabeads and subjected to different treatments for 48h. After that, the cell stimulation cocktail (PMA/Ionomycin and Golgi stop) was added for 4h, and the cells were stained for flow cytometry.

#### Studies with human T cells

For human T cells, human PBMCs were stained with anti-human CD3-PE/Cy7, anti-human CD8-FITC, and anti-human CD4-APC/Cy7 and incubated on ice for 20 min. PBMCs were then washed and stained with DAPI to distinguish live from dead cells. Live CD8^+^ T cells were sorted using fluorescence-associated cell sorting in BD FACS Aria. The isolated CD8^+^ T cells were counted using trypan blue and seeded in a 96-well plate along with human anti-CD3/CD28 magnetic beads for 48h in iMDM medium containing IL-2 (10 ng/mL). After 48h, cells were de-beaded, transferred to a 48-well plate, and allowed to proliferate. Cells were subjected to different treatments for 48h. After that, cell stimulation cocktail (PMA/Ionomycin and Golgi stop) was added for 4h, and cells were stained for flow cytometry.

#### Flow cytometry studies for macrophages and T cell cultures

For macrophages, cells were harvested and stained with Zombie Aqua Fixable Viability dye (1:100) in 1X PBS for 15 min. Cells were washed and stained with anti-F4/80-APC in FACS Buffer and incubated for 20 min at 4°C. After incubation, cells were washed and subjected to fixation and permeabilization using Cyto-fast Fix/Perm Kit according to the manufacturer’s protocol. Cells were then stained with a mixture of antibodies for intracellular markers, including anti-iNOS-PE and anti-Arg1-PE/Cy7, and incubated for 20 min at RT. Cells were washed and resuspended in FACS buffer and further acquired using FACS Symphony A5 SE (BD Biosciences, NJ, USA) and were analyzed using Flowjo v10 software. A similar protocol was used for human monocyte-derived macrophages using anti-CD86-FITC and anti-CD68-PE/Cy7 antibodies. Mouse splenic CD8^+^ T cells were stained with anti-CD8-FITC, anti-Granzyme B-PE/Dazzle 594, anti-IFN-γ-AF700 and anti-TNF-α-BV785 antibodies, and human T cells were stained with anti-Granzyme B-PE, anti-IFN-γ-APC and anti-TNF-α-PerCP/Cy5.5 antibodies.

#### RT-qPCR

Total RNA was isolated from BMDMs using TRIzol Reagent. cDNA was synthesized with the BioRAD RT Master Mix, and RT-qPCR was performed using SYBR Green Supermix according to established protocol.^74^ Relative gene expression levels were calculated using the 2^−ΔΔCt^ method. Primers are mentioned in Table S1.

#### Animal studies

The flank region of the mice was shaved before cell injection. On the day of cell injection, ∼1.5 million CT26/B16 cells suspended in 200μL FBS were injected subcutaneously in the left flank of mice, while 4T1 cells were orthotopically injected in the 3^rd^ mammary fat pad. Once tumours reached ∼50 mm^3^, mice were randomly divided into different treatment groups with an average tumor volume of ∼50 mm^3^ and subjected to different treatment regimens. Tumor volume was measured using a digital vernier caliper every other day, and calculated as (Length X Breadth^2^)/2. We excluded the mice whose tumor volume was 10 mm^3^ above or below the average tumor volume. Blinding procedures were not used for animal studies due to practical and logistical difficulties. All animal experiments were performed with 5–8 mice/group. The following formula of power calculation was employed to determine the sample size for the number of animals:

$$\text{Sample}\;\text{size}={2\;SD}^{2}{\left(Z^{\alpha /2}+Z^{\beta }\right)}^{2}/d^{2}$$

where Standard deviation = 8-10% (from previous studies)

$$Z^{\alpha /2}=Z^{0.05/2}=Z^{0.025}=1.96\left(\text{From}\;Z\;\text{table}\right)\;\text{at}\;\text{type}\;1\;\text{error}\;\text{of}\;5\%$$

$$Z^{\beta }=Z^{0.20}=0.842\;\left(\text{From}\;Z\;\text{table}\right)\;\text{at}\;80\%\;\text{power}$$

d = effect size = difference between mean values. This is kept at ∼15%; that is, if there is a minimum 15% reduction in tumor volume, the data would be significant.

Hence now formula will be, Sample size = 2 SD^2^ (1.96 + 0.842)^2^/d^2^

$$\text{Sample}\;\text{Size}=2{\left(8-10\right)}^{2}{\left(1.96+0.842\right)}^{2}/{\left(15\right)}^{2}=5-8$$

So, according to this calculation, the sample size in each group can be 5–8.

#### Anti-tumor studies

Mice were categorized into different treatment groups to evaluate the efficacy of BUP-Gel (40 mg/kg), Darifenacin-Gel (25 mg/kg), MLA-Gel (25 mg/kg), and ML375-Gel (25 mg/kg). For monotherapy studies, group 1 mice were left untreated, while group 2 received the corresponding drug-loaded hydrogel. To examine whether ACh or CARB (5 mg/kg) affected BUP-mediated tumor inhibition, mice were randomized into three groups: untreated, BUP-Gel, or BUP-ACh-Gel/BUP-CARB-Gel. For T cell depletion studies, tumor-bearing mice were divided into four groups: Untreated, BUP-Gel with Isotype IgG control antibody, BUP-Gel with anti-CD8 antibody and BUP-Gel with anti-CD4 antibody. All antibodies were administered at a dose of 250 μg/mouse, intraperitoneally on days 4 and 8 after gel injection. For comparing BUP-Gel and BUP-TS treatments, tumor-bearing mice were randomized into three groups: group 1 mice were left untreated, while group 2 and group 3 mice were treated with BUP-TS (Solution, 40 mg/kg) and BUP-Gel (40 mg/kg). For combination therapy experiments with oxaliplatin (OX), tumor-bearing mice were divided into four groups: untreated; BUP-Gel/DARF-Gel/ML375-Gel/MLA-Gel; OX alone (5 mg/kg, intravenously on days 4 and 8 after gel injection); or the combination of gel therapy and OX. Similarly, for combination with 5-Fluorouracil (5-FU), CT26 tumor-bearing mice were divided into four groups: untreated; BUP-Gel; 5-FU alone (20 mg/kg, intravenously on days 4, 8, and 12 after gel injection); or the combination of BUP-Gel with 5-FU. For combination with aCTLA4, tumor-bearing mice were randomized into four groups: untreated; BUP-Gel/DARF-Gel/ML375-Gel/MLA-Gel; aCTLA4 alone (100 μg/mouse, intraperitoneally, day 2 after gel injection); or the combination gel therapy and anti-CTLA4. For adoptive T cell therapy studies, B16-OVA tumor-bearing mice were divided into groups: groups 1 mice remained untreated, group 2 mice received 2 × 10^6^ OT-I cells via retro-orbital injection (100 μL PBS) (at the same time as group 4), while group 3 mice received BUP-Gel; group 4 mice received BUP-Gel and 2 × 10^6^ OT-I cells after 5 days of BUP-Gel injection. Combination index analysis was performed as per established study.^75^

To evaluate the effect of BUP-Gel therapy on tumor recurrence, CT26 tumors were surgically resected at ∼50 mm^3^. Mice were randomized into two groups: Untreated and BUP-Gel. BUP-Gel was injected the next day after tumor resection, and mice were observed for tumor recurrence. After 20 days of BUP-Gel therapy, CT26 cells (0.2 million cells) were injected into naive, untreated and BUP-Gel-treated mice (mice without relapse), and again, tumor growth was monitored till day 36 of initial BUP-Gel injection.

#### Toxicity studies

CT26 tumour-bearing mice were treated with different combinations of treatments as described above. At day 20, serum samples were collected and subjected to toxicity assessment, including liver function and kidney function tests.

#### 5′ single-cell RNA transcriptomics

Three CT26 tumors per group were harvested, digested, and processed to obtain a single-cell suspension. These cells were stained with anti-CD45-FITC antibody, followed by live-dead staining. Live CD45^+^ and CD45^−^ cells from the tumour were sorted and pooled in 3:1 ratio and washed thoroughly with 1X PBS. A total of 25,000 cells, pooled from untreated and treated tumors, were loaded onto separate lanes of the Chromium NextGEM Chip K. The manufacturer’s recommendations were followed to generate gene expression libraries from the captured cells in each lane. Libraries were sequenced on a NovaSeq 6000 as per the manufacturer’s instructions. Demultiplexed raw FASTQ files corresponding to gene expression libraries of each lane were mapped to the mouse reference genome GRCm39 using the 10X Genomics cellranger (v8.0.0) count pipeline. We retained 7163 and 6776 cells for the untreated and BUP-treated groups/lanes, respectively, after removing cells that did not meet our QC parameters. The QCed cells were analyzed using the R-based Seurat package (v5.0.1). Since both experiments (lanes) were conducted concurrently and ran on the same chip, no batch effects were observed, as recommended by the Seurat package. Quality-controlled cells with UMI counts greater than 100 and mitochondrial DNA content less than 20% were used to log-normalize the dataset. The most significant principal components (PCs) were used to find neighbors and to further determine clusters. A uniform manifold approximation and projection (UMAP) was applied to visualise the single-cell transcriptional profile in 2D space using the same number of most significant PCs. Markers of individual clusters (or groups) were defined as the differentially expressed genes between cells in each group and every other group, using the Wilcoxon rank-sum test implemented in the function FindAllMarkers. Genes with log-fold change >/ = 0.25 and a BH-adjusted *p* value </ = 0.05 were considered to be differentially expressed. Pairwise comparisons between the two clusters of interest were performed using the FindMarkers function with the same cutoffs as mentioned.

#### Single-cell RNA-sequencing data acquisition and analysis

Publicly available Smartseq2-based single cell RNA sequencing data and associated metadata on colorectal cancer (GSE146771) were downloaded from the TISCH website (http://tisch.comp-genomics.org/) and used for analysis with the R (v4.4.0) package Seurat (v5.3.0).^76^^,^^77^ The data were log-normalized, and highly variable genes were selected for scaling. The first twenty principal components were selected to infer cell–cell similarity and construct a shared nearest-neighbor graph. Transcriptionally distinct cell populations were identified by unsupervised clustering, and the results were visualized using Uniform Manifold Approximation and Projection (UMAP).^78^ Associated metadata, including sample information and cell-type annotations, were then incorporated into the Seurat object. Harmony (v1.2.3) was used to normalize the PCA embeddings across different patient samples.^79^ The RunHarmony function was used with the default parameters to correct batch effects with ‘Sample’ as the batch variable. The first twenty harmony dimensions and a resolution of 0.5 were then used with the FindNeighbors and FindClusters functions. Dimension reduction was performed using the RunUMAP function.

To investigate the expression landscape of cholinergic signaling components in colorectal cancer, a gene set comprising muscarinic and nicotinic acetylcholine receptor genes was curated (Table S3). Their expression was then examined across patients and annotated cell lineages in the tumor samples of the dataset using violin, feature and dot plot visualizations. Muscarinic and nicotinic receptor expression scores were first computed at the single-cell level using the *AddModuleScore* function in *Seurat v5.3.0*, based on curated receptor gene sets. These scores were then aggregated at the patient level by calculating the median module score across all cells per tumour sample, yielding patient-specific muscarinic and nicotinic receptor activity scores. For each receptor class, samples were stratified into High and Low expression groups using the cohort-wide median score as the cut-off. These group labels were appended to the Seurat metadata and used for downstream comparative analyses. Cell-type proportions were calculated by normalizing the number of cells in each annotated cluster to the total number of cells per sample. Targeted analyses were performed for selected immune populations, including M1 macrophages, exhausted CD8^+^ T cells (CD8Tex), and regulatory T cells (Tregs). Statistical comparisons between High and Low receptor groups were performed using a two-sided Wilcoxon rank-sum test implemented via the *ggpubr* v0.6.1^80^package.

#### Multivariate Cox regression and Kaplan-Meier analysis

Univariate Kaplan-Meier analysis was performed using the KM-Plotter database.^81^ The publicly available microarray gene expression data and associated metadata on colorectal cancer (GSE14333) were downloaded from the GEO database and analyzed in R (v4.5.3).^77^ The dyplr (v1.2.1) package was utilized for data organization and for the isolation of clinical covariates. The independent prognostic significance was determined by the multivariate Cox proportional hazard regression model, and the resulting data (Hazard ratios, *p*-value) were plotted using the ggforest function in the survminer(v0.5.2) package. Kaplan-Meier plots were made using the survfit function using the survival package (v3.8.6). The resulting survival curves, along with risk tables, were visualised utilising the ggsurvplot function within the survminer package (v0.5.2).The remaining plots were made using ggplot2 package (v4.0.3).^80^

#### Flow cytometry studies for mouse samples

Excised tumor sections were chopped, homogenized and were subjected to enzymatic digestion using DNase I (50U/mL) and collagenase (0.75 mg/mL) in shaker incubator at 37°C for 45 min. After digestion, cells were strained to obtain single cells.^82^ For Macrophages, single cells were stained with Zombie Aqua Fixable Viability dye (1:100) in 1X PBS for 15 min for live/dead staining. After incubation, cells were washed and stained with a cocktail of antibodies including anti-CD45-FITC, anti-Ly6G-PerCP/Cy5.5, anti-F4/80-APC, anti-CD11b-APC/Cy7, and incubated for 20 min at 4°C in the dark, followed by washing with FACS buffer. Cells were then fixed and permeabilized with Cyto-Fast Fix/Perm Kit according to manufacturer’s protocol. After fixation and permeabilization, cells were stained with a cocktail of antibodies in 100 μL Cyto-Fast Perm Wash Buffer, including anti-iNOS-PE, anti-ARG1-PE/Cy7, anti-IFN-γ-BV711 and anti-TNFα-BV785. The cells were incubated for 20 min at RT and washed with Cyto-Fast Perm Wash Buffer. Cells were resuspended in FACS Buffer and acquired using FACS Symphony A5 SE (BD Biosciences, NJ, USA) and were analyzed using Flowjo v10 software.

For T cells, the cell suspension was enriched using a Percoll gradient, and the buffy coat was collected. Cells were first stained with anti-CD4-PE/Cy7 in FACS buffer and incubated for 20 min at 4°C in the dark. Cells were washed and subjected to stimulation with PMA (50 ng/mL) and Ionomycin (1 μg/mL) in complete RPMI-1640 at 37°C for 2h, and 1X Monensin was added 2h after the addition of the stimulation cocktail to inhibit Golgi vesicle-mediated secretion of cytokines. After 4h incubation, cells were washed and stained with Zombie Aqua Fixable Viability dye (1:100) in 1X PBS for 15 min. This was followed by washing and staining of cell surface markers with a cocktail of antibodies targeting surface markers, including anti-CD8-FITC, anti-CD45-PerCP/Cy5.5, anti-CD3e-BV421, anti-PD-1-APC/Cy7, anti-TIM3-APC and anti-CTLA4. Cells were incubated at 4°C for 20 min in the dark, washed and further fixed and permeabilised using Cyto-Fast Fix/Perm kit according to the manufacturer’s protocol. Cells were further stained with a cocktail of antibodies, including anti-Granzyme B-PE/Dazzle594, anti-IFNγ-AF700 and anti-TNFα-BV785 in Cyto-Fast Perm Wash Buffer, and incubated for 20 min at RT, washed and finally resuspended in FACS Buffer. Cells were then acquired using FACS Symphony A5 SE (BD Biosciences, NJ, USA) and were analyzed using Flowjo v10 software.

For memory T cells, peripheral blood from tumour-bearing mice was collected and treated with 1X RBC lysis buffer for 10 min, followed by washing with 1× PBS at 500g for 5 min; this wash step was repeated twice to ensure complete removal of RBCs. The remaining cells were resuspended in FACS buffer for blocking, then stained with an antibody cocktail consisting of anti-CD8–FITC, anti-CD44–PE, anti-CD62L–PerCP-Cy5.5, anti-CD3e–PE-Cy7, anti-CD4–APC-Cy7, and anti-CD45–BV510, and incubated at 4°C in the dark for 20 min. After two additional PBS washes, cells were stained with DAPI for live/dead discrimination and acquired on a FACS Symphony A5 SE (BD Biosciences, NJ, USA) and were analyzed using Flowjo v10 software.

#### Patient tumor explant studies

Fresh specimens of patient-derived tumor fragments were cultured as per the published article.^48^ Briefly, 96-well plates were coated with 40 μL of extracellular matrix, which consisted of 2/3rd of matrigel and 1/3rd of collagen and were allowed to polymerize at 37°C for 30 min. Tumour fragments were washed on a cell strainer and placed one per well on top of the solidified matrix. A second 40 μL extracellular matrix layer was added, solidified, and topped up with 120 μL tumor medium. These fragments were subjected to different treatments for 48h and were processed for flow cytometry.

#### Flow cytometry studies for human explants

Tumor fragments were finely chopped and subjected to enzymatic digestion with DNase I (0.026 mg/mL) and Collagenase IV (1 mg/mL) for 1h at 37°C on a constant shaking platform. Cells were then strained and washed with cold 1X PBS to obtain a single-cell suspension. For macrophages, cells were stained with Zombie Aqua Fixable Viability dye (1:100) in 1X PBS for 15 min to detect live/dead cells. Cells were washed and stained with anti-CD45-AF700, anti-CD11b-APC/Cy7, anti-CD86-FITC, and anti-CD163-PE in FACS Buffer, incubated for 20 min at 4°C. After incubation, cells were washed and subjected to fixation and permeabilization using Cyto-fast Fix/Perm Kit according to the manufacturer’s protocol. Cells were then stained with anti-CD68-PE/Cy7 and incubated for 20 min at RT. Cells were washed and resuspended in FACS buffer and further acquired using FACS Symphony A5 SE (BD Biosciences, NJ, USA) and were analyzed using Flowjo v10 software. For T cells, the cell suspension was incubated with a cell stimulation cocktail, followed by a Golgi stop for 4h. Cells were harvested and stained with Zombie Aqua Fixable Viability dye (1:100) in 1X PBS for 15 min. Cells were washed and stained with anti-CD45-AF700, anti-CD3e-PE/Cy7, anti-CD8-FITC, and anti-CD4-APC/Cy7 in FACS Buffer, incubated for 20 min at 4°C. After incubation, cells were washed and subjected to fixation and permeabilization using Cyto-fast Fix/Perm Kit according to the manufacturer’s protocol. Cells were then stained with antibodies for intracellular markers, including anti-Granzyme B-PE, anti-IFN-γ-APC and anti-TNFα-PerCP/Cy5.5 and incubated for 20 min at RT. Cells were washed and resuspended in FACS buffer and further acquired using FACS Symphony A5 SE (BD Biosciences, NJ, USA) and were analyzed using Flowjo v10 software.

### Quantification and statistical analysis

We performed statistical analyses using GraphPad Prism 10 (GraphPad Software). Data is represented as mean ± SD or mean ± SEM as mentioned in all figure legends. We performed pairwise comparisons using unpaired or paired Student’s *t* test, multiple comparisons among groups using one-way ANOVA, and growth kinetic analysis using two-way ANOVA. Post-hoc corrections were performed using the Dunnett method for one-way ANOVA and the Tukey method for two-way ANOVA. Differences between groups were considered significant at *p*-values below 0.05 (∗*p* < 0.05, ∗∗*p* < 0.005, ∗∗∗*p* < 0.0005, and ∗∗∗∗*p* < 0.0001).

## Supplemental information

Document S1. Figures S1–S7 and Tables S1–S3

Data S1. Clinical details of the patients with colon cancer, including gender, age, stage, grade, and MMR status

Data S2. Clinical details of the GSE14333 cohort for multivariate survival analysis in patients with colon cancer

Data S3. Quantification of NEF/ChAT, CD8, and CD68 in colon cancer tissue sections

Document S2. Article plus supplemental information
