# Supplementary material for: Targeting of tripartite neuron-cancer-immune cell crosstalk augments response to chemotherapy and immunotherapy
Source: Cell Rep Med. 2026 Aug 18;7(9):102994. doi: 10.1016/j.xcrm.2026.102994 (PMC13589505; doi:10.1016/j.xcrm.2026.102994)
Supplement: Supplementary file 1 [file mmc1.pdf]

Cell Reports Medicine, Volume *7*

Supplemental information

Targeting of tripartite neuron-cancer-immune cell crosstalk augments response to chemotherapy and immunotherapy

Dolly Jain, Nishant Pandey, Somesh K. Jha, Shreya Sinha, Ragini Singh, Junaid Alam, Bharti Aggarwal, Neelam Chauhan, Arunima Acharya, Anu P.V., Vandana Dhangar, Ritika Bhargava, Nikhil K. Chourasiya, Ali Khan, Arnab K. Sahoo, Debasish Nath, Lalita Mehra, Kajal Rana, Poonam Yadav, Jahanvi Ralhan, Saroj Rajput, Asish Pal, Arindam Maitra, Santiswarup Singha, Rajesh Panwar, Satish Khurana, Ujjaini Dasgupta, Sandeep K. Bhoriwal, Jyothi S. Prabhu, Prasenjit Das, Veena S. Patil, and Avinash Bajaj

Supplementary Information

Targeting of Tripartite Neuron-Cancer-Immune Cell Crosstalk Augments Response to Chemotherapy and Immunotherapy

Dolly Jain, Nishant Pandey, Somesh K. Jha, Shreya Sinha, Ragini Singh, Junaid Alam, Bharti Aggarwal, Neelam Chauhan, Arunima Acharya, Anu P. V., Vandana Dhangar, Ritika Bhargava, Nikhil K. Chourasiya, Ali Khan, Arnab K. Sahoo, Debasish Nath, Lalita Mehra, Kajal Rana, Poonam Yadav, Jahanvi Ralhan, Saroj Rajput, Asish Pal, Arindam Maitra, Santiswarup Singha, Rajesh Panwar, Satish Khurana, Ujjaini Dasgupta, Sandeep K. Bhoriwal, Jyoti S Prabhu, Prasenjit Das, Veena S. Patil, Avinash Bajaj

Supplementary Table S1. Sequences of the primers used in the study. (Related to Figure 4)

Gene Name	Forward primer (5' to 3')	Reverse primer (5' to 3')
Nos2	GTCCAACCTGCAGGTCTTTG	GATGTTGCCATTGTTGGTGGC
Arg1	GAAACAGAGTATGACGTGAGAG	TTCATTGGCTTTCCCCACAGA
Gapdh	GGAGAAAACCTGCCAAGTATGA	GGAGTTGCTGTTGAAGTCGC

Supplementary Table S2. Table showing the combination Index and the calculations for the combination therapies studies. (Related to Figure 5, Figure 6)

Combination	Fractional Inhibition of Combination	Expected Bliss	Bliss Synergy Score	Combination Index	Interaction *p* value	Effect
BUP-Gel + OX	0.67	0.63	0.045	0.93	0.437	Additive
BUP-Gel + aCTLA4	0.77	0.76	0.005	0.99	0.369	Additive
BUP-Gel + OT-I	0.89	0.67	0.224	0.75	0.926	Additive
BUP-Gel + FU	0.71	0.58	0.126	0.82	0.998	Additive
DARF-Gel + OX	0.58	0.52	0.047	0.92	0.793	Additive
ML375-Gel + OX	0.66	0.479	0.18	0.73	0.480	Additive
MLA-Gel + OX	0.71	0.64	0.067	0.90	0.520	Additive
DARF-Gel + aCTLA4	0.55	0.47	0.077	0.86	0.992	Additive
ML375-Gel + aCTLA4	0.71	0.57	0.143	0.79	0.760	Additive
MLA-Gel + aCTLA4	0.74	0.56	0.184	0.752	0.670	Additive

Supplementary Table S3. Gene set comprising muscarinic and nicotinic acetylcholine receptors. (Related to Figure 6)

**Muscarinic Receptors**	**Nicotinic Receptors**
CHRM1	*CHRNA1*
CHRM2	*CHRNA2*
CHRM3	*CHRNA3*
CHRM4	*CHRNA4*
CHRM5	*CHRNA5*
	CHRNA6
	CHRNA7
	CHRNA8
	CHRNA9
	CHRNA10
	CHRNB1
	CHRNB2
	CHRNB3
	CHRNB4
	CHRND
	CHRNE
	CHRNG

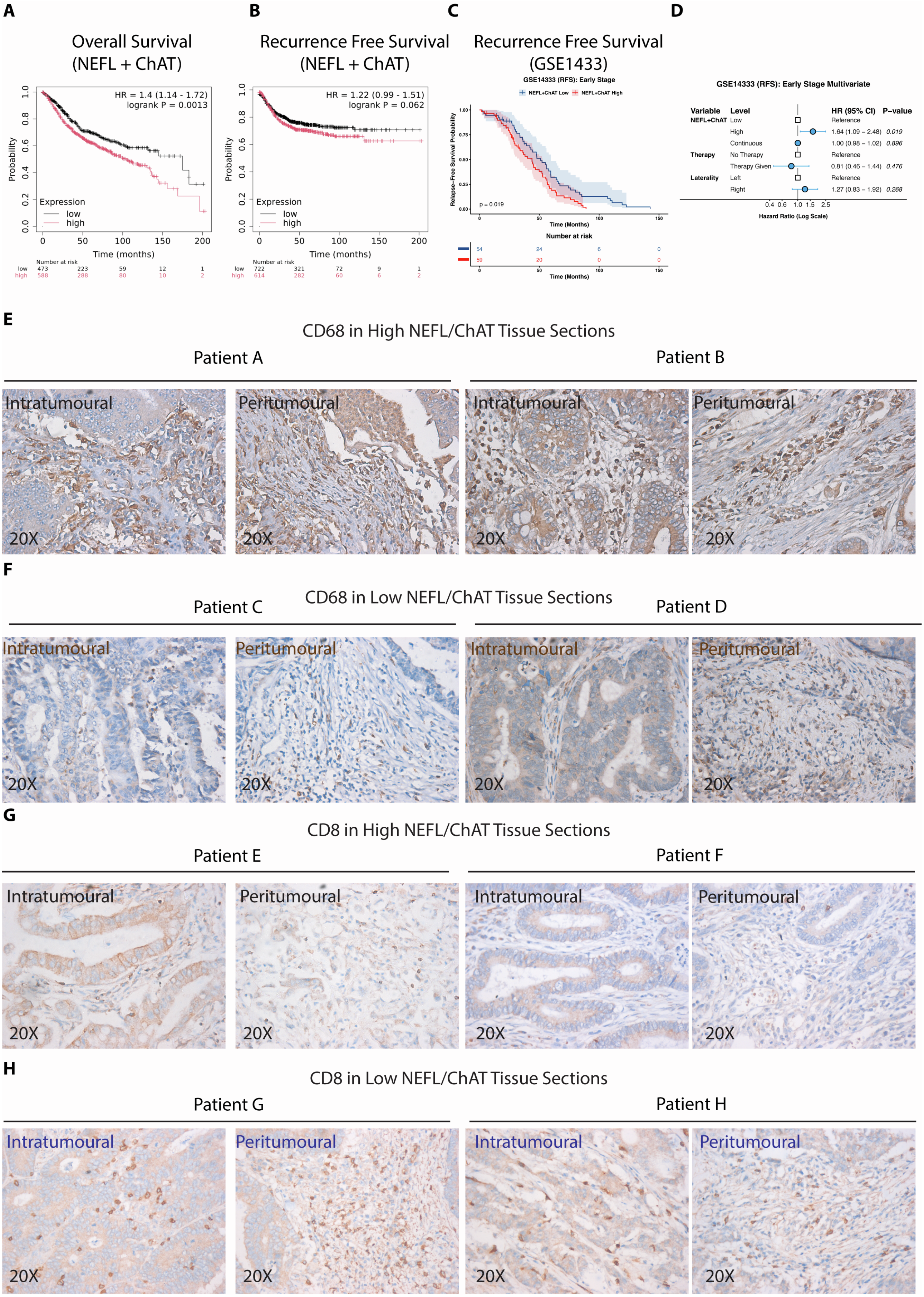
A
Overall Survival
(NEFL + ChAT)
HR = 1.4 (1.14 - 1.72)
logrank P = 0.0013
Probability
Expression
low
high
Time (months)
Number at risk
low 473 223 59 12 1
high 588 288 80 10 2
B
Recurrence Free Survival
(NEFL + ChAT)
HR = 1.22 (0.99 - 1.51)
logrank P = 0.062
Probability
Expression
low
high
Time (months)
Number at risk
low 722 321 72 9 1
high 614 282 60 6 2
C
Recurrence Free Survival
(GSE1433)
GSE14333 (RFS): Early Stage
NEFL+ChAT Low
NEFL+ChAT High
Relapse-Free Survival Probability
p = 0.019
Time (Months)
Number at risk
54 24 6 0
59 20 0 0
Time (Months)
D
GSE14333 (RFS): Early Stage Multivariate
Variable Level HR (95% CI) P-value
NEFL+ChAT Low Reference
High 1.64 (1.09 – 2.48) 0.019
Continuous 1.00 (0.98 – 1.02) 0.896
Therapy No Therapy Reference
Therapy Given 0.81 (0.46 – 1.44) 0.476
Laterality Left Reference
Right 1.27 (0.83 – 1.92) 0.268
Hazard Ratio (Log Scale)
E
CD68 in High NEFL/ChAT Tissue Sections
Patient A
Patient B
Intratumoural
Peritumoural
20X
F
CD68 in Low NEFL/ChAT Tissue Sections
Patient C
Patient D
G
CD8 in High NEFL/ChAT Tissue Sections
Patient E
Patient F
H
CD8 in Low NEFL/ChAT Tissue Sections
Patient G
Patient H

Supplementary Figure S1. High NEFL/ChAT expression is associated with poor survival, increased macrophage infiltration, and reduced CD8 T cell infiltration. (Related to Figure 1)

(A) Kaplan-Meier analysis showing overall survival of patients with colon cancer with high (n = 588) or low (n = 473) expression of both NEFL and ChAT in the KM-Plotter database.

(B) Kaplan-Meier analysis showing Recurrence-free survival of patients with colon cancer with high (n = 614) or low (n = 722) levels of both NEFL and ChAT expression in the KM-Plotter database.

(C) Kaplan-Meier analysis showing recurrence-free survival of patients with colon cancer with high and low expression of NEFL and ChAT in the GSE14333 dataset.

(D) Forest plot showing multivariate Cox regression analysis of NEFL and CHAT expression in the GSE14333 early-stage colon cancer cohort, with hazard ratios (HRs), *P* value and 95% confidence intervals.

(E, F) Representative IHC images of tissue sections excised from patients with colon cancer with high (E) and low (F) NEFL/ChAT expression, stained for $CD68^+$ cells, show increased infiltration of $CD68^+$ cells in the high-NEFL/ChAT sections.

(G, H) Representative IHC images of tissue sections excised from patients with colon cancer with high (G) and low (H) NEFL/ChAT expression, stained for $CD8^+$ T cells, suggest the increased infiltration of CD8 cells in the intratumoural and peritumoural regions in the sections with low NEFL/ChAT expression.

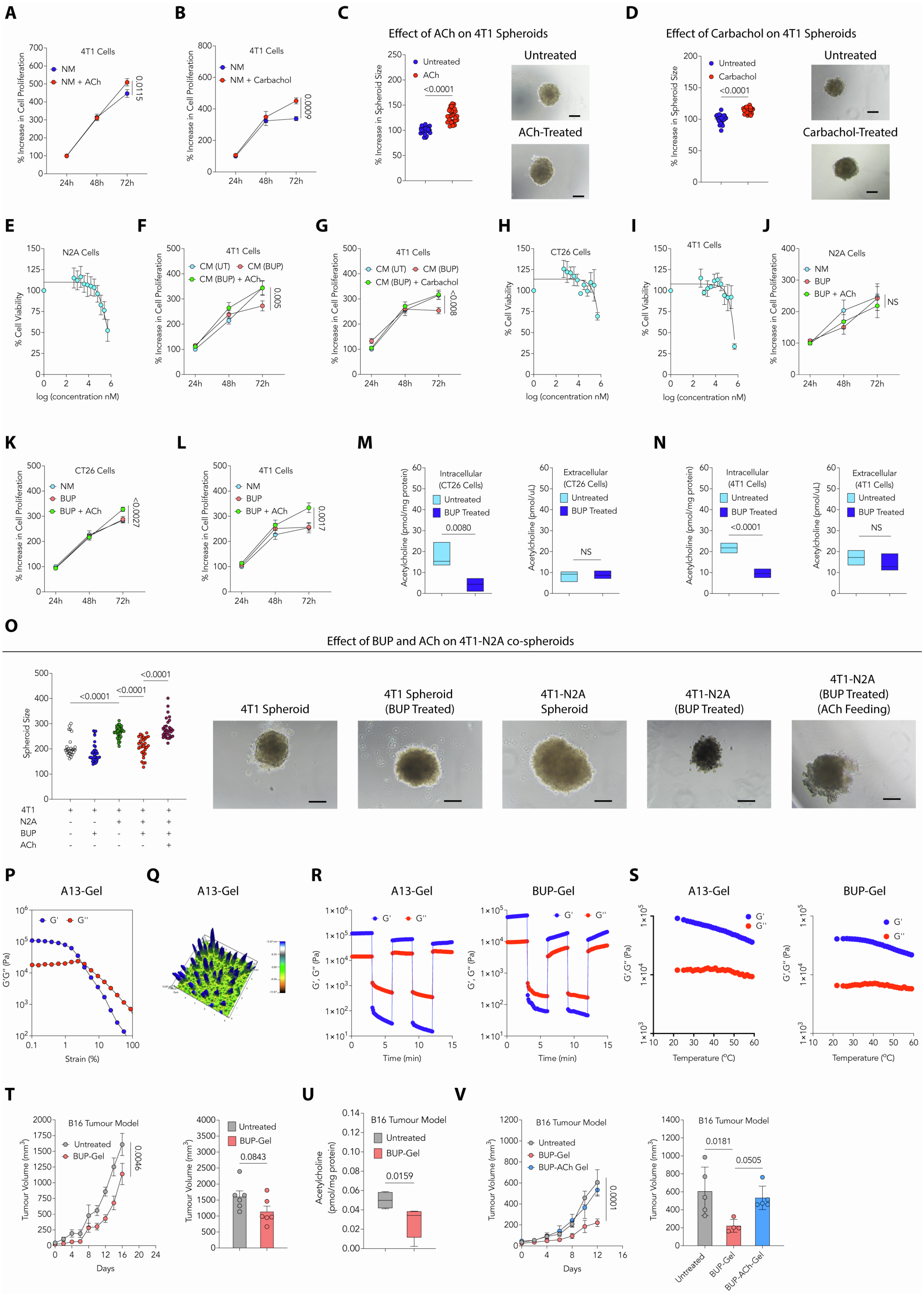
A
4T1 Cells
NM
NM + ACh
% Increase in Cell Proliferation
0.0115
24h
48h
72h
B
4T1 Cells
NM
NM + Carbachol
% Increase in Cell Proliferation
0.0009
C
Effect of ACh on 4T1 Spheroids
Untreated
ACh
<0.0001
% Increase in Spheroid Size
Untreated
ACh-Treated
D
Effect of Carbachol on 4T1 Spheroids
Untreated
Carbachol
<0.0001
% Increase in Spheroid Size
Untreated
Carbachol-Treated
E
N2A Cells
% Cell Viability
log (concentration nM)
F
4T1 Cells
CM (UT)
CM (BUP)
CM (BUP) + ACh
0.005
% Increase in Cell Proliferation
G
4T1 Cells
CM (UT)
CM (BUP)
CM (BUP) + Carbachol
<0.008
H
CT26 Cells
% Cell Viability
log (concentration nM)
I
4T1 Cells
% Cell Viability
log (concentration nM)
J
N2A Cells
NM
BUP
BUP + ACh
NS
% Increase in Cell Proliferation
K
CT26 Cells
NM
BUP
BUP + ACh
<0.0027
L
4T1 Cells
NM
BUP
BUP + ACh
0.0017
M
Intracellular (CT26 Cells)
Untreated
BUP Treated
0.0080
Acetylcholine (pmol/mg protein)
Extracellular (CT26 Cells)
Untreated
BUP Treated
NS
Acetylcholine (pmol/uL)
N
Intracellular (4T1 Cells)
Untreated
BUP Treated
<0.0001
Acetylcholine (pmol/mg protein)
Extracellular (4T1 Cells)
Untreated
BUP Treated
NS
Acetylcholine (pmol/uL)
O
Effect of BUP and ACh on 4T1-N2A co-spheroids
Spheroid Size
<0.0001
<0.0001
<0.0001
4T1
N2A
BUP
ACh
4T1 Spheroid
4T1 Spheroid (BUP Treated)
4T1-N2A Spheroid
4T1-N2A (BUP Treated)
4T1-N2A (BUP Treated) (ACh Feeding)
P
A13-Gel
G'
G''
G'G'' (Pa)
Strain (%)
Q
A13-Gel
R
A13-Gel
BUP-Gel
G', G'' (Pa)
Time (min)
S
A13-Gel
BUP-Gel
G', G'' (Pa)
Temperature (°C)
T
B16 Tumour Model
Untreated
BUP-Gel
0.0046
Tumour Volume (mm³)
Days
Untreated
BUP-Gel
0.0843
U
B16 Tumour Model
Untreated
BUP-Gel
0.0159
Acetylcholine (pmol/mg protein)
V
B16 Tumour Model
Untreated
BUP-Gel
BUP-ACh Gel
0.0001
Tumour Volume (mm³)
Days
B16 Tumour Model
0.0181
0.0505
Untreated
BUP-Gel
BUP-ACh-Gel

Supplementary Figure S2. BUP inhibits cell proliferation and tumour growth by inhibiting neuronal ACh release. (Related to Figure 2)

(A, B) Change in cell proliferation (Mean ± SEM, n = 4) of 4T1 cells on treatment with ACh (A) and carbachol (B) at different timepoints.

(C, D) Percentage increase in spheroid size (Mean ± SEM, n = 3) and representative images of 4T1 spheroids on treatment with ACh (C) and carbachol (D) (scale bar = 50 μm).

(E) Cytotoxicity (Mean ± SEM, n = 3) of BUP against N2A cells.

(F, G) Change in cell proliferation (Mean ± SEM, n = 4) of 4T1 cells on treatment with conditioned media of untreated N2A cells (CM (UT)), BUP-treated N2A cells (CM (BUP)), and ACh- or carbachol-fed BUP-treated N2A cells.

(H, I) Cytotoxicity (Mean ± SEM, n = 3) of BUP against CT26 (H) and 4T1 (I) cells.

(J-L) Change in cell proliferation (Mean ± SEM, n = 3) of N2A (J), CT26 (K) and 4T1 (L) cells on treatment with BUP and BUP along with ACh.

(M, N) Quantification of intracellular and extracellular released ACh (Mean ± SEM, n = 5-6) from BUP-treated CT26 (M) and 4T1 (N) cells.

(O) Spheroid size (Mean ± SEM, n = 3) and representative images of N2A-4T1 co-spheroids under different treatment conditions (scale bar = 50 μm).

(P) Amplitude sweep characterisation of A13-Gel.

(Q) AFM image of A13-Gel.

(R) Thixotropic characterisation of A13-Gel and BUP-Gel evaluating the self-healing property and structural stability.

(S) Temperature sweep oscillation characterisation of A13-Gel and BUP-Gel.

(T, U) Tumour growth kinetics (Mean ± SEM, n = 6) (T) and ACh quantification (U) (Mean ± SEM, n = 3) in B16 tumours on treatment with BUP-Gel.

(V) Tumour growth kinetics (Mean ± SEM, n = 5) and final day tumour volume of B16 tumours on treatment with BUP-Gel and ACh-loaded BUP-ACh-Gel.

Two-tailed Student's t test for Figures S2A-S2D, S2M, S2N, S2T, S2U. One-way ANOVA with Dunnett's post-hoc test for Figures S2F, S2G, S2J-S2L, S2V. Two-way ANOVA with Tukey's post-hoc test for Figures S2T, S2U *$p < 0.05$, **$p < 0.001$, and ****$p < 0.0001$ for the indicated comparisons.

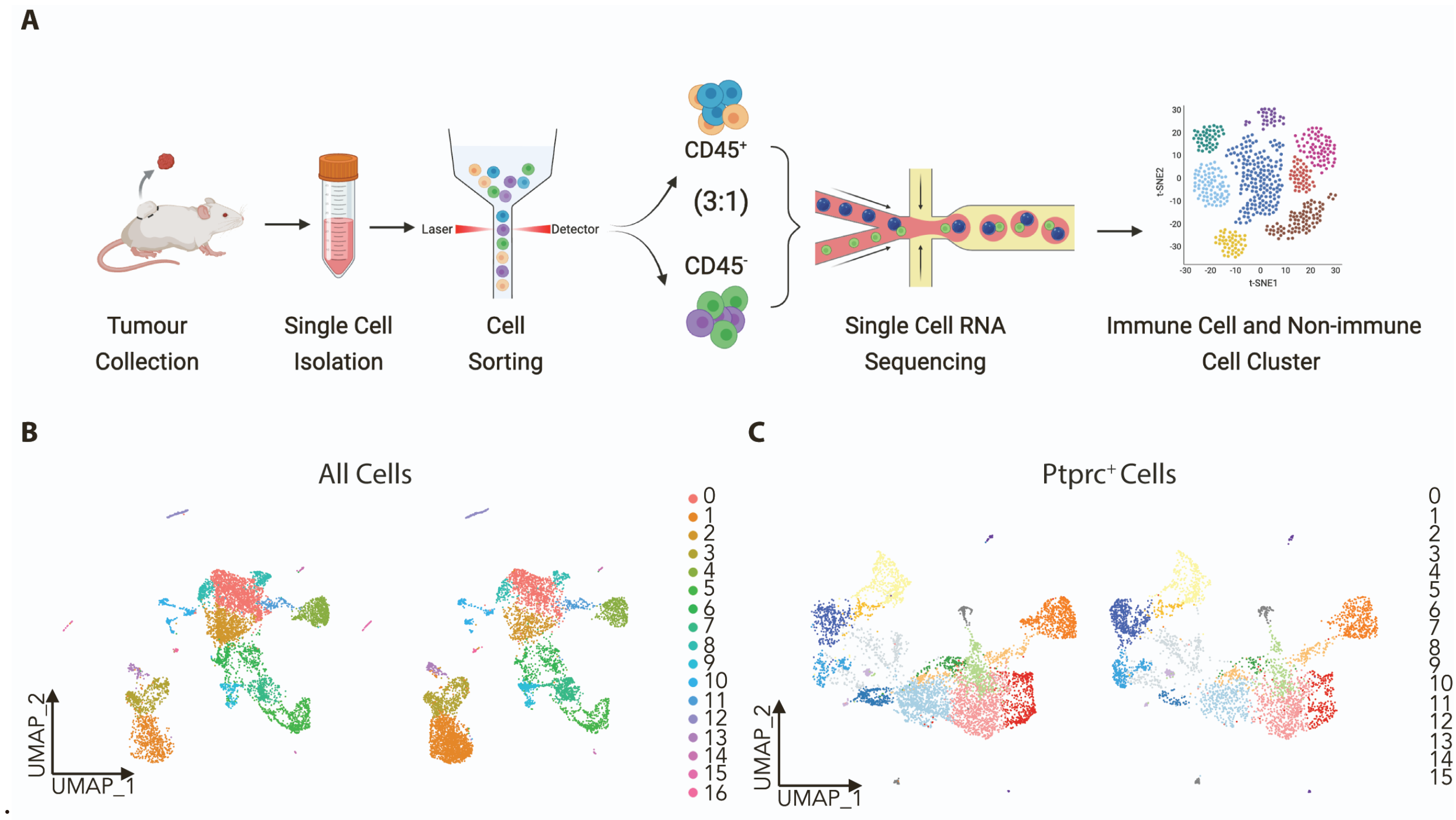

Supplementary Figure S3. Single-cell RNA analysis reveals BUP-Gel-mediated immune cell reprogramming. (Related to Figure 3)

(A) Schematic showing the strategy starting from the isolation and sorting of cells from tumours and subjecting them to a single-cell RNA seq platform using the 10X genomics platform.

(B) The UMAP plot showing the sixteen distinct clusters of all cells from UT and BUP-treated groups.

(C) The UMAP plot shows sixteen distinct clusters of all immune cells re-clustered from *Ptprc*$^{+}$ cells of UT and BUP-treated groups.

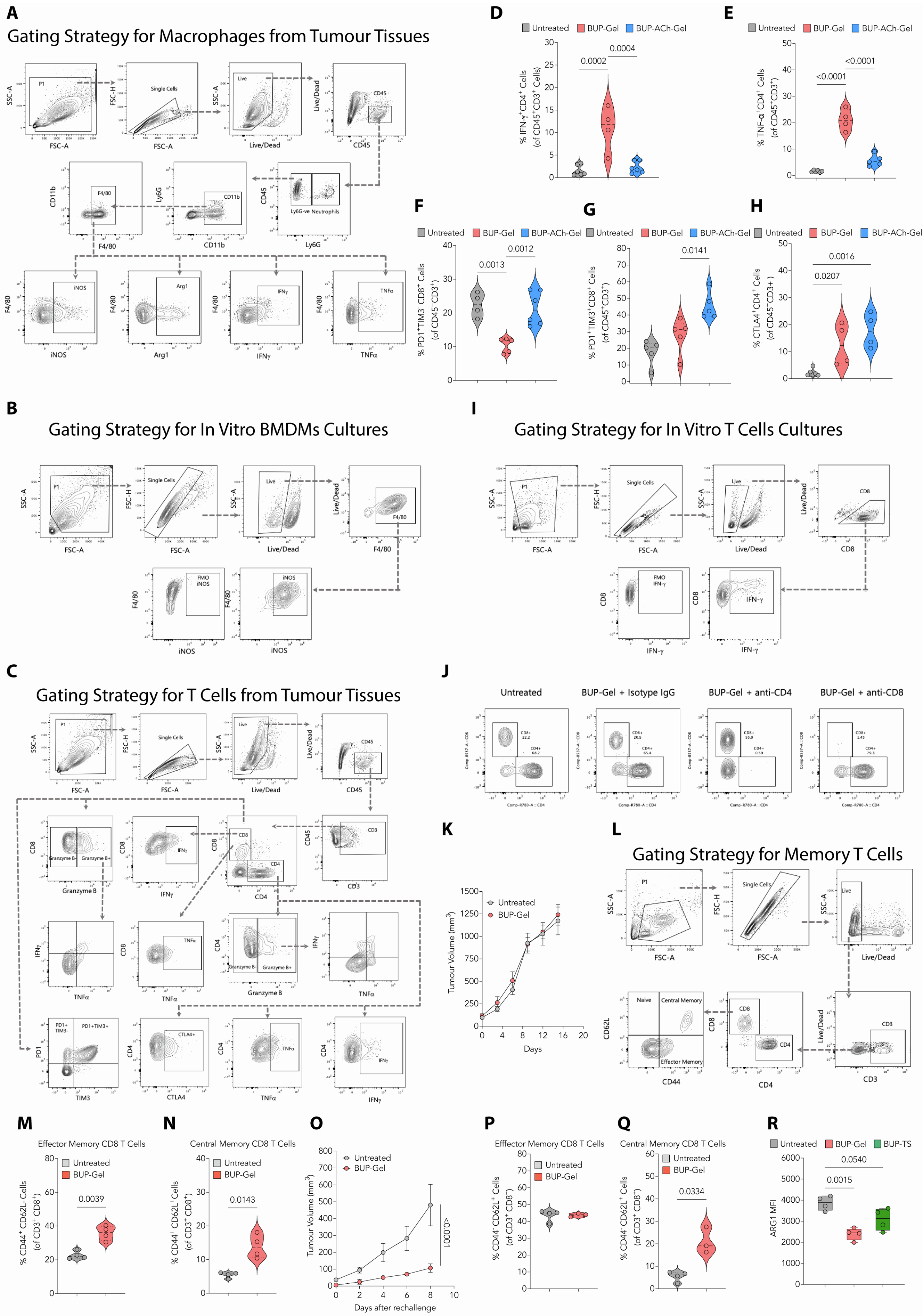
A
Gating Strategy for Macrophages from Tumour Tissues
B
Gating Strategy for In Vitro BMDMs Cultures
C
Gating Strategy for T Cells from Tumour Tissues
D
E
F
G
H
I
Gating Strategy for In Vitro T Cells Cultures
J
Untreated
BUP-Gel + Isotype IgG
BUP-Gel + anti-CD4
BUP-Gel + anti-CD8
K
L
Gating Strategy for Memory T Cells
M
Effector Memory CD8 T Cells
N
Central Memory CD8 T Cells
O
P
Effector Memory CD8 T Cells
Q
Central Memory CD8 T Cells
R
Untreated
BUP-Gel
BUP-ACh-Gel
BUP-TS

Supplementary Figure S4. BUP-Gel activates anti-tumour immunity. (Related to Figure 4)

(A) Gating strategy used to quantify the different populations of macrophages in tumour tissues on different treatments.

(B) Gating strategy used to quantify macrophages in BMDMs on different treatments.

(C) Gating strategy used to quantify the different populations of T cells in tumour tissues on different treatments.

(D-H) Changes (Mean ± SEM, n = 4-6) in IFN-γ^+CD4$^+$ (D), TNF-α^+CD4$^+$ (E), PD1$^+$TIM3$^+$CD8$^+$ (F), PD1$^+$TIM3$^-$CD8$^+$ (G) and CTLA4$^+$CD4$^+$ (H) in untreated, BUP-Gel-treated, and BUP-ACh-Gel-treated CT26 tumours.

(I) Gating strategy used to quantify cytotoxic markers on T cells in splenic CD8$^+$ T cells on different treatments.

(J) Flow cytometry showing the depletion of CD4 and CD8 T cells in the blood of tumour-bearing mice.

(K) Tumour growth kinetics (Mean ± SEM, n = 6) of CT26 tumours on treatment with BUP-Gel in immunocompromised NSG mice.

(L) Gating strategy used to quantify the different memory T cells in peripheral blood in tumour-bearing mice on different treatments.

(M, N) Changes (Mean ± SEM, n = 4) in effector memory (CD44$^+$CD62L$^-$CD8$^+$) T cells (M) and central memory (CD44$^+$CD62L$^-$CD8$^+$) T cells (N) in the blood of untreated and BUP-Gel-treated CT26 tumour-bearing mice.

(O) Tumour growth kinetics (Mean ± SEM, n = 3-4) of CT26 tumours after rechallenge of CT26 cells in mice, showing no recurrence of tumours after resection in BUP-Gel-treated mice.

(P, Q) Changes (Mean ± SEM, n = 3-4) in effector memory (CD44$^+$CD62L$^-$CD8$^+$) (P) and central memory (CD44$^+$CD62L$^-$CD8$^+$) T cells (Q) in the blood of mice rechallenged with CT26 tumours at day 36.

(R) Percentage change in the population (Mean ± SEM, n = 4) of MFI of ARG1 within macrophages in untreated, BUP-Gel-treated, and BUP-TS-treated CT26 tumours.

One-way ANOVA with Dunnett's post-hoc test for Figures S4D-S4H, S4M, S4N, S4R, and Two-tailed Student's t test for Figures S4M, S4N, S4P, S4Q. Two-way ANOVA with Tukey's post-hoc test for Figures S4K, S4O. *$p < 0.05$, **$p < 0.001$, and ****$p < 0.0001$ for the indicated comparisons.

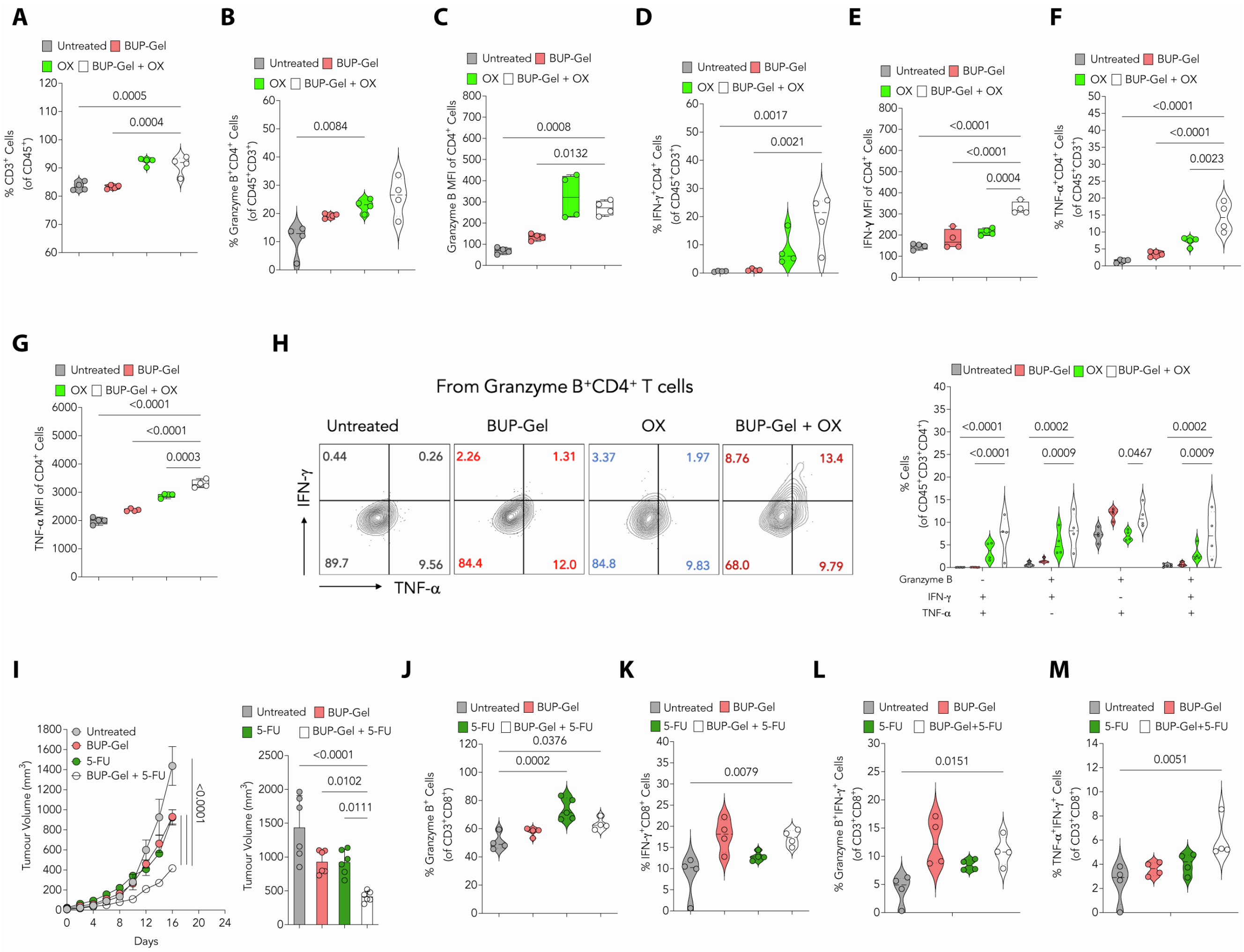

Supplementary Figure S5. BUP-Gel therapy improves outcomes of chemotherapy and promotes anti-tumour response. (Related to Figure 5)

(A-F) Change in the population (Mean ± SEM, n = 4) of $CD3^+$ (A), Granzyme B^+CD4^+ and its MFI (B, C), IFN-γ^+CD4^+ and its MFI (D, E), TNF-α^+CD4^+ and its MFI (F, G) in untreated, BUP-Gel-treated, OX-treated and tumours treated with the combination of BUP-Gel and OX.

(H) Flow cytometry plots (H) and percentage change (I) in the $CD4^+$ populations (Mean ± SEM, n = 4) co-expressing Granzyme B, IFN-γ or TNF-α.

(I) Tumour growth kinetics and final day tumour volume (Mean ± SEM, n = 6) of CT26 tumours on treatment with BUP-Gel, 5-FU, and a combination of BUP-Gel with 5-FU.

(J-M) Change in the population (Mean ± SEM, n = 4) of Granzyme B^+CD8^+ (J), IFN-γ^+CD8^+ (K), Granzyme B^+IFN-γ^+CD8^+ (L) and TNF-α^+IFN-γ^+CD8^+ (M) cells in untreated, BUP-Gel-treated, 5-FU-treated and tumours treated with the combination of BUP-Gel and 5-FU.

One-way ANOVA with Dunnett's post-hoc test for Figures S5A-S5H, S5J-S5M and Two-way ANOVA with Tukey's post-hoc test for Figure S5I. *$p < 0.05$, **$p < 0.001$, and ****$p < 0.0001$ for the indicated comparisons.

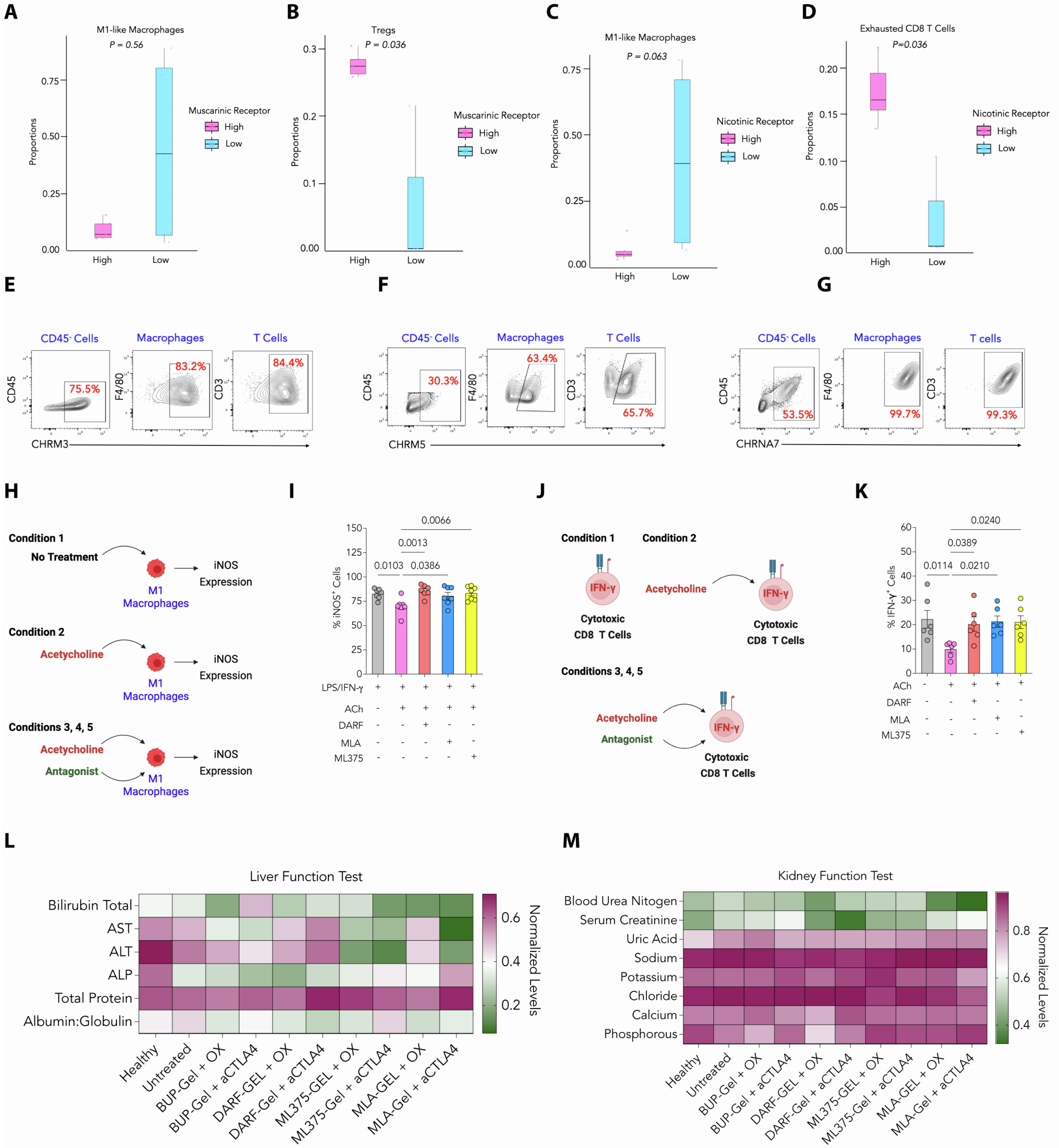

Supplementary Figure S6. Specific ACh receptor blockade reverses ACh-mediated immunosuppression. (Related to Figure 6)

(A-D) Box plots representing proportions of M1-like macrophages (A) and Tregs (B) within high vs low muscarinic receptor expression, as well as M1-like macrophages (C) and exhausted CD8 T cells (CD8 Tex) (D) within high vs low nicotinic receptor expression.

(E-G) Flow cytometry plots showing the expression of CHRM3 (E), CHRM5 (F) and CHRNA7 (G) in $CD45^-$ cancer cells, $F4/80^+$ macrophages and $CD3^+$ T cells.
(H) Schematic of the study plan to decipher the effects of DARF, ML375, and MLA on M1-like macrophages.
(I) Percentage change in $iNOS^+$ macrophages (Mean ± SEM, n = 7) of M1 macrophages treated with DARF (1 μM), ML375 (1 μM) and MLA (5 μM) in the presence of ACh (50 μM).
(J) Schematic showing the study plan to decipher the effect of DARF, ML375 and MLA on cytotoxic CD8 T cells.
(K) Percentage change in IFN-$\gamma^+$$CD8^+$ T cells (Mean ± SEM, n = 6) treated with DARF (1 μM), ML375 (1 μM) and MLA (5 μM) in the presence of ACh (150 μM).
(L, M) Toxicity studies showing the changes in LFT (L) and KFT (M) parameters (Normalized levels, n = 5-6) in healthy and tumour-bearing mice on different treatments.
One-way ANOVA with Dunnett's post-hoc test for Figures S6I, S6K. *p < 0.05, **p < 0.001, and ****p < 0.0001 for the indicated comparisons.

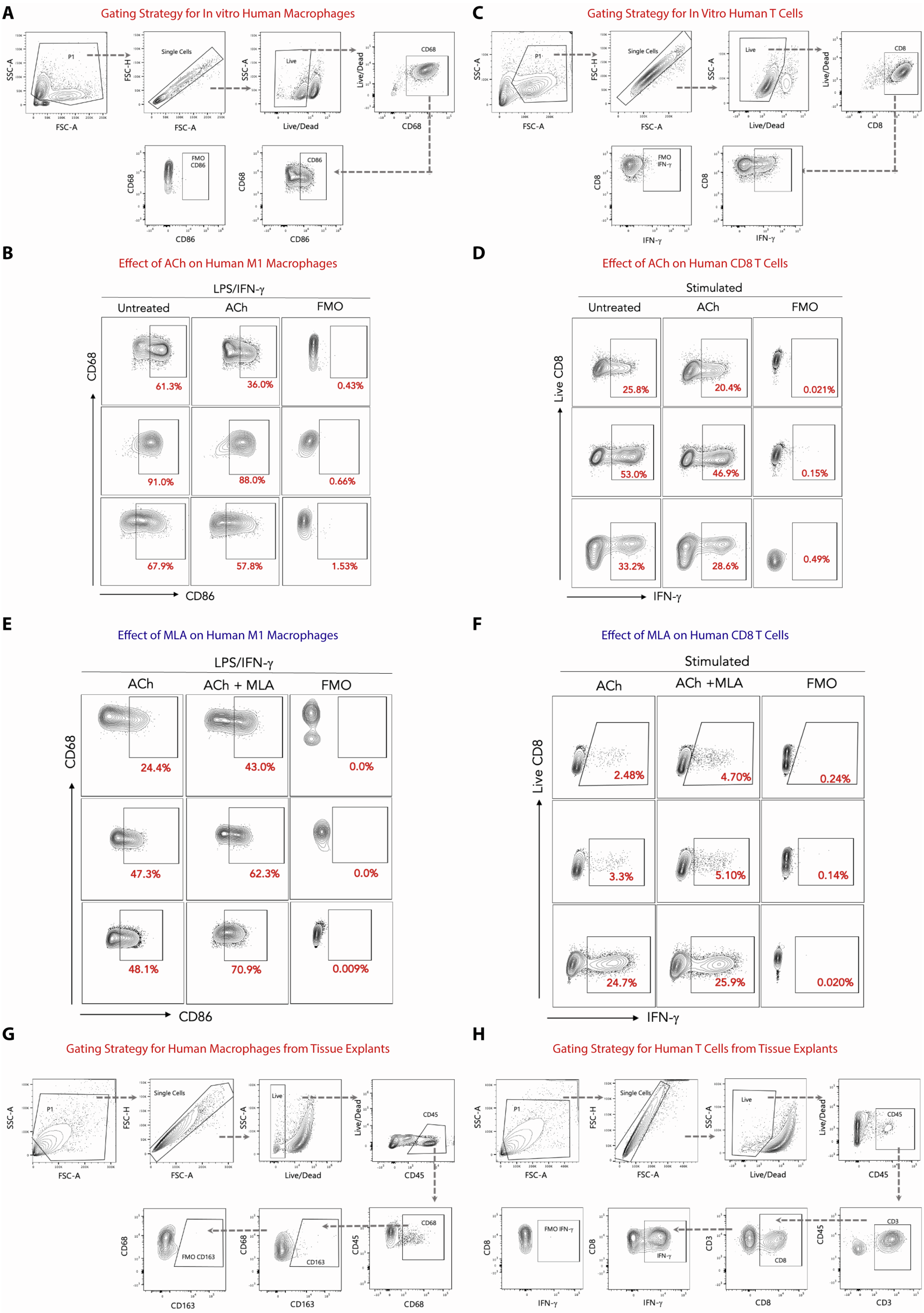
A
Gating Strategy for In vitro Human Macrophages
P1
Single Cells
Live
CD68
SSC-A
FSC-A
FSC-H
Live/Dead
CD68
FMO CD86
CD86
C
Gating Strategy for In Vitro Human T Cells
P1
Single Cells
Live
CD8
FMO IFN-γ
IFN-γ
B
Effect of ACh on Human M1 Macrophages
LPS/IFN-γ
Untreated
ACh
FMO
CD68
CD86
61.3%
36.0%
0.43%
91.0%
88.0%
0.66%
67.9%
57.8%
1.53%
D
Effect of ACh on Human CD8 T Cells
Stimulated
Untreated
ACh
FMO
Live CD8
IFN-γ
25.8%
20.4%
0.021%
53.0%
46.9%
0.15%
33.2%
28.6%
0.49%
E
Effect of MLA on Human M1 Macrophages
LPS/IFN-γ
ACh
ACh + MLA
FMO
CD68
CD86
24.4%
43.0%
0.0%
47.3%
62.3%
0.0%
48.1%
70.9%
0.009%
F
Effect of MLA on Human CD8 T Cells
Stimulated
ACh
ACh +MLA
FMO
Live CD8
IFN-γ
2.48%
4.70%
0.24%
3.3%
5.10%
0.14%
24.7%
25.9%
0.020%
G
Gating Strategy for Human Macrophages from Tissue Explants
P1
Single Cells
Live
CD45
CD68
CD163
FMO CD163
H
Gating Strategy for Human T Cells from Tissue Explants
P1
Single Cells
Live
CD45
CD3
CD8
FMO IFN-γ
IFN-γ

Supplementary Figure S7. CHRNA7 blockade reverses ACh-mediated immune modulation in human immune cells and patient-derived tumour explants. (Related to Figure 7)

(A) Gating strategy used to assess $CD68^+$ human macrophages derived from monocytes, which are sorted from human PBMCs and subjected to different treatments.

(B) Flow cytometry plots along with FMO controls showing the impact of ACh on CD86 expression in $CD68^+$ cells in human monocyte-derived M1-like macrophages.

(C) Gating strategy used to assess $CD8^+$ T cells sorted from human PBMCs and subjected to different treatments.

(D) Flow cytometry plots along with FMO controls showing the effect of ACh on IFN-γ expression in $CD8^+$ cells sorted from human PBMCs.

(E) Flow cytometry plots along with FMO controls showing the effect of MLA (CHRNA7 antagonist) on CD86 expression in $CD68^+$ cells in ACh-treated human monocyte-derived M1-like macrophages.

(F) Flow cytometry plots along with FMO controls showing the effect of MLA (CHRNA7 antagonist) on IFN-g expression in $CD8^+$ cells in ACh-treated $CD8^+$ T cells.

(G) Gating strategy used to assess $CD68^+$ macrophages in patient-derived tumour fragments on different treatments.

(H) Gating strategy used to assess $CD8^+$ T cells in patient-derived tumour fragments on different treatments.
